# Thermo-Electro-Mechanical Vibration Analysis of Microbeams with Non-Ideal Boundary Conditions

**DOI:** 10.3390/mi17091028

**Published:** 2026-08-28

**Authors:** Sıdıka Nur Yardim, Saim Kural

**Affiliations:** Department of Mechanical Engineering, Manisa Celal Bayar University, Sehit Prof. Dr. Ilhan Varank Campus, Yunusemre, 45110 Manisa, Turkey; saim.kural@cbu.edu.tr

**Keywords:** microbeam, MEMS, thermal effect, method of multiple scales, microbeams under temperature influence, non-ideal boundary conditions, Monte Carlo simulation

## Abstract

In this study, the vibration characteristics of microbeams subjected to thermal loads and electric field effects under non-ideal boundary conditions were investigated. The non-ideal boundary conditions are modeled as a linear combination of ideal fixed and simply supported boundary conditions. The weighting factor, k, is defined as a measure of deviation from the ideal boundary conditions. To determine the influence of the boundary conditions, both non-ideal fixed and non-ideal simply supported beams were examined. The equations of motion of the system are derived using Hamilton’s principle, and the Method of Multiple Scales, a perturbation technique, is applied to obtain approximate analytical solutions for both linear and nonlinear vibration responses. The effects of thermal loads, electric field influence, and non-ideal boundary conditions on the natural frequencies were investigated. The results demonstrate that the natural frequencies vary significantly with changes in the weighting factor, k. To address operational reliability, a formal probabilistic sensitivity and uncertainty analysis using Monte Carlo simulations is integrated, quantifying how uncertainties in geometrical and thermal parameters propagate to structural instability. Furthermore, a theoretical framework for calibrating the abstract boundary parameter via Finite Element Model updating is introduced. These results indicate that idealized boundary condition assumptions may lead to significant inaccuracies in the design and reliability assessment of micro-electromechanical systems (MEMSs) operating under the combined effects of thermal and electric fields. Relying solely on idealized support assumptions may result in significant failure (damage) predictions owing to the underestimation of thermally induced buckling and electrical pull-in risks arising from assembly imperfections. Furthermore, a probabilistic sensitivity analysis is implemented to quantify how parameter uncertainties propagate through the system, bridging the gap between pure deterministic modeling and operational reliability.

## 1. Introduction

Microsystems are defined as systems with dimensions between 1 µm and 1 mm. Due to technological developments in recent years, a significant shift toward miniaturization has been observed in scientific research. The utilization of small-scale systems is increasingly favored, particularly in heat and mass transfer applications, to achieve higher processing speeds and improved performance [1].

Size effects at the micron and nanometer scales cannot be adequately captured by classical elasticity theories because they are devoid of an internal material length-scale parameter. Therefore, the Modified Couple Stress Theory (MCST) is commonly applied in the analysis of microsystems. Park and Gao [2] investigated the characteristics of Euler–Bernoulli microbeams using the MCST. The discrepancy between the results of the classical beam theory and the applied theory becomes significant when the beam thickness is very small. Ma et al. [3] developed a Timoshenko beam model using MCST. Yang et al. [4] defined a material-specific parameter, known as the material length scale parameter (l), in the MCST framework they employed. Atci and Bağdatlı [5] investigated the vibrations of a fluid-conveying microbeam with non-ideal boundary conditions using a boundary condition model.

In recent years, the vibration characteristics of micro- and nanostructures under thermal loading have been investigated by numerous researchers in light of this theory. For example, Younis and Nayfeh [6] investigated the response of resonant microbeams subjected to electrical activation with a nonlinear model that accounts for mid-plane stress and electrostatic forces. Using the multi-timescale perturbation method, they validated the system stability and the effects of the design parameters on the frequency response curves with experimental results. Nayfeh et al. [7] obtained numerical solutions using the finite difference method with the Galerkin method to analyze the nonlinear dynamics of a resonant gas sensor consisting of a cantilever microbeam with a microplate attached to its end and electrostatic driving.

Akkoca et al. [8] investigated the linear vibration behavior of ceramic microbeams located in an electric field and supported by a central support using modified stress couple theory. The multi-time scale and perturbation methods were used to solve the equation of motion. The effects of the support position and size on the natural frequencies and mode shapes were considered. Akbaş [9] investigated the post-buckling behavior of functionally graded beams under temperature influence using Timoshenko beam theory and two- and three-dimensional continuum models. He examined the effects of temperature-dependent material properties and geometric nonlinear conditions on the critical buckling load and stress distribution. Yıldırım [10] discussed the control of vibrations in microbeams subjected to magnetoelectric charge using piezoelectric actuators and the maximization principle. The solutions implemented in the MATLAB environment demonstrated the effectiveness and robustness of the applied control method in minimizing the kinetic energy of the microbeam. Li et al. [11] investigated the free vibration of functional graded material (FGM) beams with piezoelectric layers bonded to their surface, subjected to both temperature increase and voltage in a thermal environment. Using geometrically nonlinear equations based on the Euler–Bernoulli theory, they investigated the effects of thermal-electro-mechanical loading on the frequency responses of the system. Warminska et al. [12] investigated the dynamic behavior of composite beams subjected to thermal and mechanical loading using the extended Timoshenko beam model and showed that an increase in the ambient temperature has a decisive effect on the beam response. Hamza-Cherif et al. [13] investigated the vibration analysis of nanobeams under thermal effects in non-ideal Euler–Bernoulli beam models under different boundary conditions using the Differential Transformation Method (DTM). Zhang et al. [14] analyzed the free vibration of Euler–Bernoulli beams in a uniform thermal environment using bi-Helmholtz core two-phase ideal/non-ideal mixed integral models.

Ahmed and Marin [15] developed a novel thermoelastic model utilizing the MCST for Euler–Bernoulli nanobeams characterized by temperature-dependent properties. Majid and Nevvab [16] analyzed the thermal vibration of a rotating functionally graded Timoshenko microbeam in thermal environments under different temperature distributions based on MCST. Mamu and Karatoğun [17] analytically investigated the vibrational behavior of nanobeams resting on a nonlinear basis in a thermal environment using both the MCST and the multiple scales method (a perturbation technique). Andrianov et al. [18] comprehensively evaluated the nonlinear oscillations generated by electrical stimulation, considering geometric nonlinearity within the Kirchhoff model framework. He investigated the dependence of the tensile value on the geometric nonlinearity. Eiadtrong and Wattanasakulpong [19] included the effects of classical and nonclassical boundary conditions on system dynamics in the thermal vibration analysis of functionally graded porous beams using a modified Fourier series method.

In line with the latest theoretical developments, the complex effects of thermal effects and size-dependent parameters on microsystems are being investigated. Abouelregal et al. [20] investigated the thermomechanical behavior of flexible microbeams subjected to electrical stress and sinusoidal heat pulses. In their study, based on a modified double stress theory and a non-Fourier heat transfer model, the Laplace transform was used in closed-form solutions, and the effects of electrical and thermal parameters on the dynamic response of the system were compared with those of conventional models. Lastly, Chang and Zhang [21] described the transverse vibrations of disordered Bernoulli–Euler beams with elastic boundary conditions and subjected to thermal effects using a two-phase local/non-local integral model. Analyses performed using the Generalized Differential Quadrature Method (GDQM) showed that the boundary spring stiffness directly affects the frequency of the system and that neglecting non-local thermal effects causes deviations in the calculated frequency values.

In the structural design and precise determination of the stability limits of micro-electromechanical systems (MEMSs), realistic and physically accurate modeling of boundary conditions is extremely critical. Although traditional studies generally assume ideal clamped or simply supported boundary conditions, actual boundary conditions always lie between these two ideal cases due to manufacturing tolerances, assembly defects, and support flexibilities. Wu and Chang [22] investigated the effects of general and flexible boundary conditions on axially loaded beams in detail using elastic spring elements placed at the boundary points. Lee [23], on the other hand, proposed weighted boundary condition formulations to present these non-ideal relaxations and flexibilities in boundary conditions in a more compact and analytical framework. In this study, a continuously varying weighting factor (k) was defined and included in the theoretical model to characterize non-ideal support defects and flexibilities.

When designing practical micro-electromechanical systems (MEMSs), achieving perfect clamped or ideal simple supports is generally unfeasible owing to defects inherent to the assembly process, boundary compliance, and the nature of manufacturing tolerances. The presence of such non-ideal boundary conditions significantly alters the stiffness of the structure, making it highly susceptible to thermal buckling and electrical pull-in under the influence of multiphysical field loadings. Therefore, relying on idealized boundary assumptions when attempting to predict the operational limits of device behavior is highly risky and should be avoided.

This study aims to fill this research gap by conducting a comprehensive investigation of microbeam dynamics under the influence of electrical and thermal loads, considering the effects of nonideal boundary conditions. To provide a novel perspective on this problem, this research work accounts for the effects of boundary conditions through a continuously varying weighting factor, k, which represents the non-perfection of the microbeam’s boundary condition. By utilizing the method of multiple scales, the impacts of the interplay between nonideal boundaries, electric fields, and thermal loads on both linear and nonlinear dynamics were analyzed.

## 2. Materials and Methods

### 2.1. Derivation of the Equations of Motion

The kinetic and potential energy expressions for the microbeam system under thermal loading are analyzed in this section. These energy expressions are utilized to derive the equations of motion of the system using Hamilton’s principle. It is assumed that the microbeam operates under an axial thermal load and is subjected to a given temperature difference (ΔT) and thermal expansion (α). During the motion of the microbeam, the axial (longitudinal) displacement is denoted by u*(x, t), whereas the transverse displacement is represented by w*(x, t). The kinetic and potential energies of the system, including the thermal loads, are expressed as Equation (1) and Equation (2), respectively.

(1) $$T=\frac{1}{2}\rho_{K}A_{K}\overset{L}{\underset{0}{\int }}\left\{\left.{\dot{w}}^{*2}+{\dot{u}}^{*2}\right\}\right.dx^{*}$$

(2) $$\begin{array}{l} V=\frac{1}{2}E_{K}A_{K}\overset{L}{\underset{0}{\int }}{\left(u^{*\prime }+\frac{1}{2}w^{*\prime 2}\right)}^{2}dx^{*}+\frac{1}{2}E_{K}I_{K}\overset{L}{\underset{0}{\int }}w^{*\prime \prime 2}dx^{*}+\\ \overset{L}{\underset{0}{\int }}N\left(u^{*\prime }+\frac{1}{2}w^{*\prime 2}\right)dx^{*}+\frac{1}{2}G_{K}A_{K}l^{2}\overset{L}{\underset{0}{\int }}w^{*\prime \prime 2}dx^{*}+\frac{1}{2}\int_{0}^{L}\alpha EA\Delta T(w^{*\prime 2})dx^{*}=0 \end{array}$$

Equation (1) presents the kinetic energy expression resulting from the transverse and longitudinal vibrations of the beam. The first term in Equation (2) represents the longitudinal extension of the microbeam during transverse vibration, and E_K_A_K_ stands for the axial stiffness. The second term is the flexural energy representing the bending effect, which stems from the bending of the beam. Here, E_K_I_K_ stands for the flexural rigidity. The third term is the potential energy originating from the tensile force, i.e., the axial force, applied to the microbeam. The fourth term accounts for the size effect due to the micro-scale dimensions of the beam, representing the Modified Couple Stress Theory. Finally, the last term is the strain energy induced by the temperature effect, namely the thermal loading. In these expressions, A_K_ represents the cross-sectional area, ρ_K_ refers to the density, and L is the length of the microbeam. The modulus of elasticity and the area moment of inertia are designated by E_K_ and I_K_, respectively. Additionally, N represents the axial force, G_K_ the shear modulus, and l the material length scale parameter.

In Equation (3) the equations of motion for the system are formulated by applying Hamilton’s principle finalized in Equation (4).

(3) $$-\rho_{k}A_{k}{\ddot{w}}^{*}-E_{k}I_{k}w^{*iv}-G_{k}A_{k}l^{2}w^{*iv}+Nw^{*\prime \prime }+\alpha EA\Delta Tw^{*\prime \prime }+E_{k}A_{k}(u^{*\prime \prime }w^{*\prime }+w*\prime \prime u^{*\prime }+\frac{3}{2}{\left(w^{*\prime }\right)}^{2}w^{*\prime \prime })=0$$

(4) $$-\rho_{k}A_{k}{\ddot{u}}^{*}+E_{k}A_{k}(u^{*\prime \prime }+w^{*\prime }w^{*\prime \prime })=0$$

To obtain a general and simplified solution independent of the material and geometry of the beam, a non-dimensionalization process is applied to the equations of motion. The resulting dimensionless parameters are as in Equation (5).

(5) $$\begin{array}{l} \varepsilon {\bar{\alpha }}_{1}=-\frac{\varepsilon_{0}rL^{2}}{Nd^{3}},\;\;\;\varepsilon {\bar{\alpha }}_{2}=\frac{E_{k}A_{k}}{N}\frac{d^{2}}{L^{2}}={V_{1}}^{2}\frac{d^{2}}{L^{2}},\;\;\;{\bar{N}}_{T}=\alpha \Delta T{V_{1}}^{2}\\ V_{1}=\sqrt{\frac{E_{k}A_{k}}{N}},\;\;\;\;\;v_{f2}=\frac{E_{k}I_{k}}{NL^{2}},\;\;\;\;\;\;\;\gamma^{2}=\frac{G_{k}A_{k}l^{2}}{NL^{2}} \end{array}$$

By simplifying the derived equations of motion, the non-dimensional equation of motion is obtained in the form of Equation (6), independent of material properties and system geometry.

(6) $$\ddot{w}-w^{\prime \prime }+(v_{f2}+\gamma^{2})w^{ıv\prime }+\mu \dot{w}+N_{T}w^{\prime \prime }=\varepsilon {\bar{\alpha }}_{1}{V_{el}}^{2}(t)(1+2w)+{\bar{\alpha }}_{2}[\int_{0}^{1}w^{\prime 2}dx]w^{\prime \prime }$$

N_T_ is the thermal efficiency coefficient, V_f_ is the coefficient of beam and γ is the coefficient of microbeam. α_1_ is the electro-static force parameter and α_2_ is the coefficient of beam elasticity.

### 2.2. Perturbation Analysis

The Method of Multiple Scales, which is one of the perturbation techniques, is used to obtain approximate analytical solutions for the equation of motion. In this method, the time variable is decomposed into two distinct scales: the fast time scale *T*_0_ = *t* and the slow time scale *T*_1_ = *εt*. The temporal derivative operators are subsequently expanded as ∂/∂*t* = $D_{0}+\varepsilon D_{1}\;$ and $\partial^{2}$/∂$t^{2}$ = ${D_{0}}^{2}+2\varepsilon {D_{0}D}_{1}$. For the transverse displacement (*w*), acting as the dependent variable of the system, a two-term perturbation series expansion is employed in the form of (*w* = *w*_0_ + *εw*_1_). When the defined expansions for time, displacement, and velocity are substituted into the equation of motion and terms with the same powers of ε are collected, the system is reduced into two solvable sub-equations at the orders of *O*(1) and *O*(**ε**), representing the linear and nonlinear problems, respectively. Equation (7) represents the equation of order *O*(1) and Equation (8) represents the equation of order *O*(**ε**).

(7) $$O(1):{D_{0}}^{2}w_{0}+(N_{T}-1){w_{0}}^{\prime \prime }+({v_{f}}^{2}+\gamma^{2}){w_{0}}^{ıv}=0$$

(8) $$\begin{array}{l} O\left(\varepsilon \right):{D_{0}}^{2}w_{1}+({v_{f}}^{2}+\gamma^{2}){w_{1}}^{ıv}+(N_{T}-1){w_{1}}^{\prime \prime }=\\ -2D_{0}D_{1}w_{0}-\mu D_{0}w_{0}+{\bar{\alpha }}_{2}(\int_{0}^{1}{w_{0}}^{\prime 2}dx){w_{0}}^{\prime \prime }+{\bar{\alpha }}_{1}{V_{AC}}^{2}cos^{2}\Omega_{2}T_{0}(1+2w_{0}) \end{array}$$

### 2.3. Determination of Boundary Conditions

Non-ideal boundary conditions refer to elastic (imperfect) support configurations that depart from idealized fixed or simply supported responses. In practical micro-electromechanical systems (MEMSs), while the transverse displacement at the boundary is completely restricted due to the chemical or physical bonding of the anchor to the substrate, the boundaries often permit small-scale rotations or moment-carrying capacity.

To satisfy the mature physical boundary compliance models, this behavior is fundamentally represented by equivalent springs. Since the translational spring stiffness is assumed to be infinitely rigid (K_ω_→∞) at the boundaries (e.g., x = 0), it dictates that Y(0) = 0. However, rotational compliance (imperfection) exists and is modeled via a dimensionless torsional spring of stiffness K_θ_ at the boundary, where the moment–rotation relation dictates:

(9) $$\;\;\;\;\;\;\;\;Y^{\prime \prime }\left(0\right)\pm K_{\theta }Y^{\prime }\left(0\right)=0\;\;\;\;\;\;\;\;$$

To seamlessly integrate this physical reality into the analytical solutions without encountering numerical singularities (e.g., when K_θ_→∞ for completely rigid clamps), this study utilizes a weighted boundary condition formulation based on the accepted Lee [23] model. This behavior is modeled in dimensionless form as a linear combination of the ideal fixed and ideal simply supported conditions, utilizing a weighting factor (k), as Equation (10).

(10) $${Y\left(x\right)=0\;\;\;\;\;\;\;\;\;kY}^{\prime \prime }\left(x\right)\pm \left(1-k\right)Y^{\prime }\left(x\right)=0\;\;\;\;\;\;\;\;0\leq k\leq 1$$

By rewriting the formulation in Equation (9), the exact analytical bridge between the abstract k factor and the physical torsional stiffness K_θ_ becomes clearly visible:

(11) $$Y^{\prime \prime }\left(x\right)\pm \left(\frac{1-k}{k}\right)Y^{\prime }\left(x\right)=0\;\;\;\;\;\;\;$$

Comparing these two expressions reveals the mathematical mapping between J. Lee’s weighting factor and the classical spring model:

(12) $$K_{\theta }=\left(\frac{1-k}{k}\right)\;↔\;\left(\frac{1}{1+K_{\theta }}\right)\;\;\;$$

This formulation perfectly covers the entire spectrum of boundary conditions:

- Ideal Clamped Support: When K_θ_→∞ (completely rigid clamped support), the weighting factor approaches k→0.
- Ideal Simply Supported: When K_θ_→0 (zero rotational stiffness), the weighting factor approaches k→1.
- Non-Ideal Support: Intermediate values (0 < k < 1) map precisely to finite physical rotational compliance (0 < K_θ_ < ∞).

Thus, J. Lee’s k formulation is a compact, mathematically elegant transformation of the mature spring-boundary model. Within the perturbation analysis framework, the weighting factor k represents the transition between these boundaries. Assigning a small value close to zero such as ε to the factor k defines a non-ideal clamped boundary condition. Similarly, setting this factor to (1 − ε) indicates a non-ideal simply supported condition. By substituting these coefficients into the general relationship, the boundary condition expressions for the system at orders O(1) and O(ε) are derived.

In Case (1), while the left end of the microbeam is designed as a non-ideal simple support, the right end is ideally simply supported. In Case (2), both ends of the microbeam are considered to be non-ideal simple supports. In Case (3), the left end of the microbeam is taken as a non-ideal fixed support, while the right end is ideally fixed. Finally, in Case (4), both ends of the microbeam are considered to have non-ideal fixed boundary conditions. The selected weighting factors were substituted into the equations in accordance with the nature of the perturbation algorithm, thus systematically integrating support imperfections into the solutions of the O(1) linear and O(ε) nonlinear problems. By applying this scaling to the boundary equations and decomposing the transverse displacement function into orders (*w* = *w*_0_ + ε*w*_1_), the boundary conditions for all four cases under investigation are obtained as Table 1.

### 2.4. Calibration and Physical Justification of the Weighting Factor k

The non-ideal boundary parameter k is mathematically treated as an abstract linear combination of ideal fixed and simply supported conditions. While this linear simplification introduces a degree of model uncertainty, it serves as a critical mathematical bridge to represent unquantified manufacturing tolerances, assembly compliance, and micro-friction at the supports. From a metrological perspective, to translate this abstract factor into practical applicability, k must be experimentally calibrated. The proposed model framework allows this parameter to be mapped to real-world boundary compliance using Finite Element Model (FEM) updating techniques or Bayesian calibration frameworks. By comparing the analytical frequency response curves derived in this study with experimental vibration data, the exact magnitude of k for a specific MEMS fabrication batch can be probabilistically identified, thus minimizing model uncertainty.

### 2.5. Probabilistic Uncertainty Propagation Framework

To bridge the gap between deterministic analytical modeling and operational reliability, a formal uncertainty propagation framework is implemented in this study. Standard deterministic parametric sweeps alone are insufficient to quantify how the intrinsic uncertainties associated with physical variables affect the system’s response. Therefore, a probabilistic sensitivity analysis is conducted by assigning probability density functions (e.g., Gaussian distributions with a 5% standard deviation) to critical input parameters: the material length scale parameter (l), the beam coefficient (v_f_), and the temperature difference (Δ*T*). A Monte Carlo simulation (MCS) approach is utilized to propagate these geometric manufacturing tolerances and thermal fluctuations through the derived analytical model. This methodology rigorously identifies which parameter exerts the highest influence on premature thermal buckling and structural instability.

### 2.6. Solution of the Linear Problem

In perturbation analysis, the first-order equations and boundary conditions constitute the linear problem. The solution to the equation is written in complex form shown as Equation (13).

(13) $$w_{0}(x,T_{0},T_{1})=A(T_{1})e^{i\omega T_{0}}Y(x)+\bar{A}(T_{1})e^{-i\omega T_{0}}\bar{Y}(x)$$

where A is the complex amplitude and w_0_ is the natural frequency. The time-independent function Y(x) is obtained by substituting Equation (13) into Equation (7). The solution for Y(x) is given as Equation (14).

(14) $$Y(x)=c_{1}e^{ir_{1}x}+c_{2}e^{ir_{2}x}+c_{3}e^{ir_{3}x}+c_{4}e^{ir_{4}x}=c_{1}(e^{ir_{1}x}+\frac{c_{2}}{c_{1}}e^{ir_{2}x}+\frac{c_{3}}{c_{1}}e^{ir_{3}x}+\frac{c_{4}}{c_{1}}e^{ir_{4}x})$$

The obtained equation of dispersion is organized as Equation (15).

(15) $$r_{n4}(v_{f2}+\gamma^{2})+(N_{T}-1){r_{n}}^{2}-\omega^{2}=0$$

where n = 1, 2, 3 and 4. The roots of this expression provide the r values. Since obtaining these values symbolically is not straightforward, the calculation is performed numerically.

### 2.7. Solution of the Nonlinear Problem

The equation of order O(ε) given in Equation (8) forms the nonlinear problem. The solution of this problem is given as Equation (16).

(16) $$w_{0}(x,T_{0},T_{1})=A(T_{1})e^{i\omega T_{0}}Y(x)+A(T_{1})e^{-i\omega T_{0}}\bar{Y}(x)$$

Equation (16) is substituted into Equation (8) and the solvability equation given in Equation (17) was formed. The frequency of change of the electrical coercive force is assumed to be non-zero and twice the natural frequency of the system.

(17) $$D_{1}A-k_{1}A^{2}\bar{A}+Ak_{2}+\mu AS_{1}=0$$

The third term of the solvability condition occurs through the non-ideal boundary conditions of the system. This term is obtained for four different cases of boundary conditions. For a microbeam non-ideal simply supported at both ends, the equivalents of the coefficients defined in Equation (17) for our model are as in Equations (18)–(20)

(18) $$k_{1}={\bar{\alpha }}_{2}\left\{\frac{\left(\int_{0}^{1}\bar{Y}(x)\bar{Y^{\prime \prime }}(x)\int_{0}^{1}Y^{\prime }{\left(x\right)}^{2}dxdx+2\int_{0}^{1}\bar{Y}(x)Y^{\prime \prime }(x)\int_{0}^{1}Y^{\prime }(x)\bar{Y^{\prime }}(x)dxdx\right)}{2(i\omega \int_{0}^{1}\bar{Y}(x)Y(x)dx)}\right\}$$

(19) $$S_{1}=\frac{i\omega \int_{0}^{1}Y(x)\bar{Y}(x)dx}{2(i\omega \int_{0}^{1}\bar{Y}(x)Y(x)dx)}$$

(20) $$k_{2}=({v_{f}}^{2}+\gamma^{2})k\left\{\frac{Y^{\prime }(1)\bar{Y^{\prime }}(1)+Y^{\prime }(0)\bar{Y^{\prime }}(0)}{2(i\omega \int_{0}^{1}\bar{Y}(x)Y(x)dx)}\right\}$$

Assuming the right end of the investigated microbeam is an ideal simple support, only the *k*_2_ parameter will vary as in Equation (21) due to the non-ideal boundary condition.

(21) $$k_{2}=({v_{f}}^{2}+\gamma^{2})k\left\{\frac{Y^{\prime }(0)\bar{Y^{\prime }}(0)}{2(i\omega \int_{0}^{1}\bar{Y}(x)Y(x)dx)}\right\}$$

Amplitudes in complex form are given as in Equation (22):

(22) $$A=\frac{1}{2}ae^{i\theta }$$

Amplitudes are substituted into the solvability equation given in Equation (17) and the amplitude-phase modulation equations in Equations (23) and (24) are obtained.

(23) $$a^{\prime }=-ak_{2R}-\mu aS_{1}$$

(24) $$\theta^{\prime }=\frac{1}{4}a^{2}k_{1}-k_{2I}$$

where the subscripts I and R denote the imaginary and real parts of the terms. The damping term with the damping coefficient µ is taken as equal to zero to see the amplitudes explicitly. Otherwise, the displacement of the microbeam will vanish within the time through the effect of damping. Thus, the amplitude and phase are found as shown in Equations (25) and (26).

(25) $$a(T_{1})=a_{0}e^{-k_{2R}T_{1}}$$

(26) $$\theta (T_{1})=\left(\frac{1}{4}a^{2}k_{1}-k_{2I}\right)T_{1}+\theta_{0}$$

Nonlinear natural frequency of the system is obtained as Equation (27).

(27) $$\omega_{nl}=\omega +\varepsilon \left(\frac{1}{4}k_{1}a^{2}-k_{2I}\right)$$

## 3. Numerical Solutions and Results

In this study, a microbeam system under thermal effects with non-ideal fixed and non-ideal simply supported boundary conditions is examined. The effects of non-ideal boundary conditions on the natural frequency of the system were clearly observed. The beam coefficient and microbeam coefficient were taken as *v_f_* = 0.1 and *γ* = 0.0534, respectively. The ambient temperature difference is set to Δ*T* = 250 K, which is considered to generate a thermal axial load of *N_t_* = 50.

Within the framework of the linear problem, the roots *r*_1_, *r*_2_, *r*_3_ and *r*_4_ of Equation (15) were numerically evaluated, and the boundary conditions in Equation (12) were incorporated into Equation (17). Based on the resulting equations, a coefficient matrix referred to as [K] was formulated. The calculated *r_n_* values were substituted into the coefficient matrix to determine the natural frequency *ω*, which equates the determinant of the matrix to zero. These procedures were repeated to characterize the effects of the weighting factor k, thermal loads, and other system parameters on the natural frequencies.

**Figure 1:**
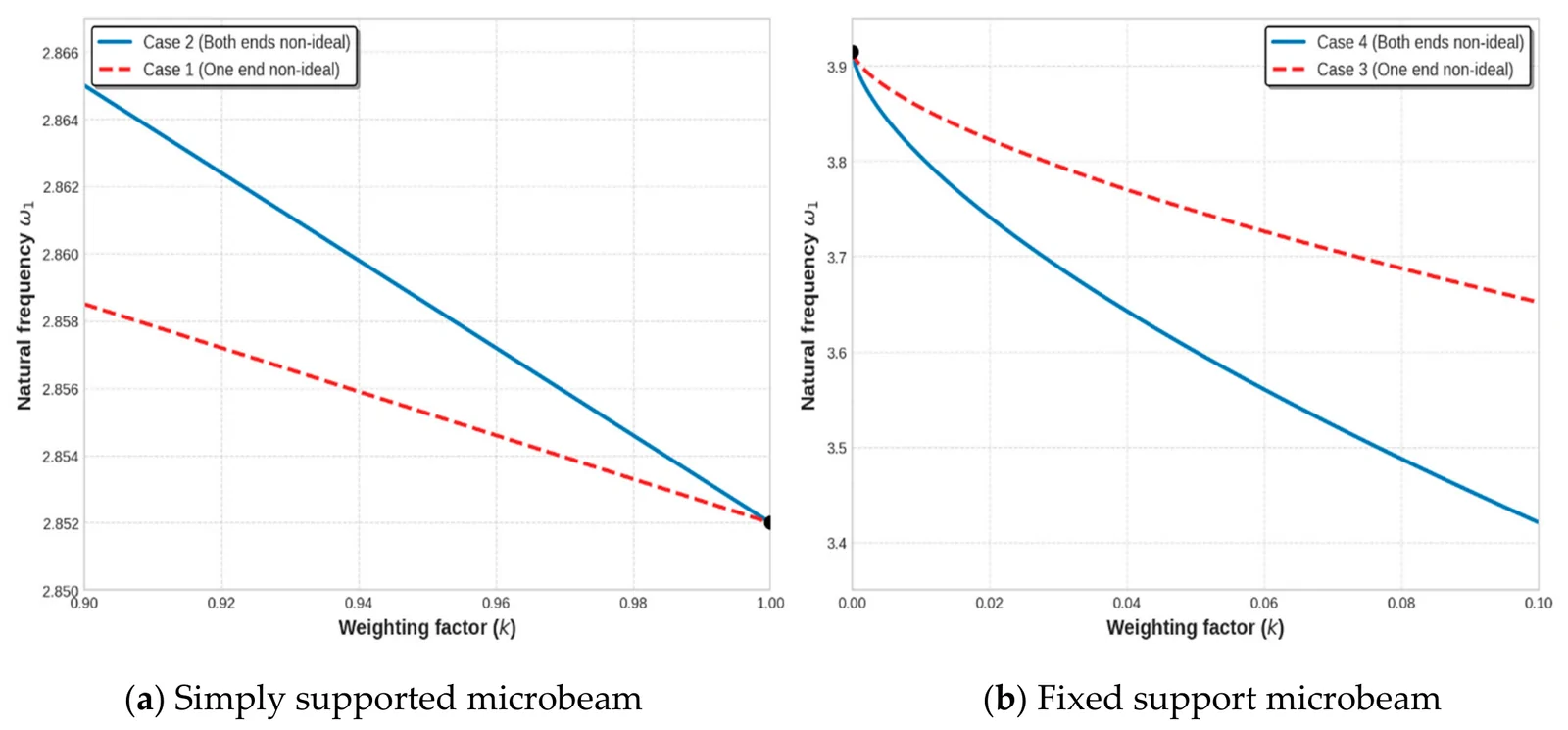
First-mode natural frequencies varying with the weighting factor for simply supported and fixed support microbeams.

Figure 1a,b present the first-mode natural frequencies varying with the weighting factor k for simply supported and fixed microbeams under thermal effects, respectively. A comparison with ideal boundary conditions is also illustrated in these figures. Figure 1a illustrates the effect of deviations from ideal support conditions (weighting factor *k*) on the fundamental natural frequency of microbeams subjected to an ambient temperature difference of Δ*T* = 250 K. It was observed that the specified temperature difference generates a substantial axial compressive force on the order of *N_t_* = 50, which significantly reduces the reference ideal frequency values compared to their room temperature counterparts.

Upon examination of the graph, it is observed that supports designed as non-ideal at both ends, represented by Case 2 and Case 4, increase frequency deviations compared to the singly non-ideal configurations represented by Cases 1 and 3. In Figure 1b, it is observed that while the beam undergoes thermally induced axial softening, the addition of a k = 0.10 relaxation at the fixed support leads to a total collapse of system stiffness, causing the frequency curve to shift toward the buckling instability region.

**Figure 2:**
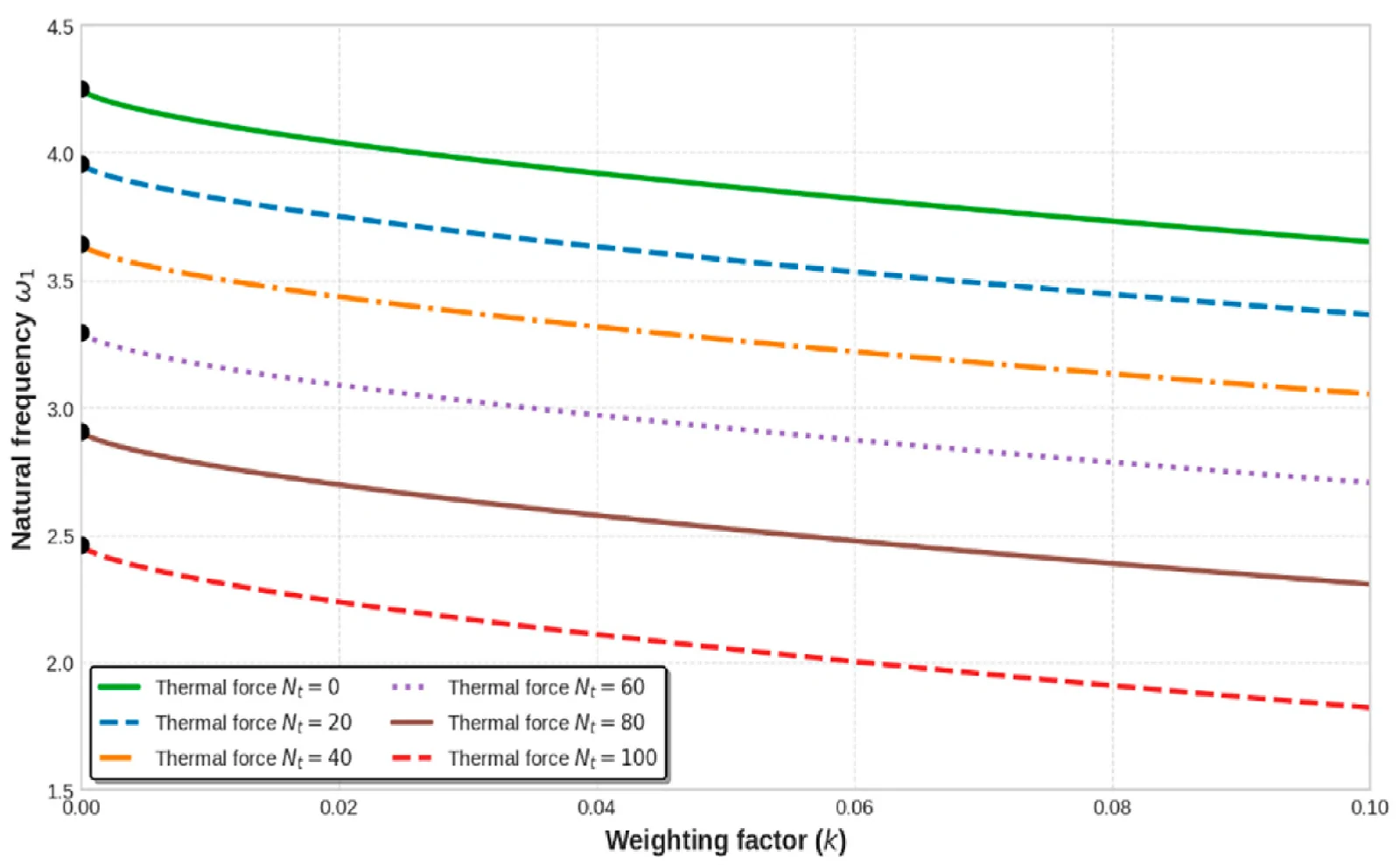
Natural frequencies of the first-mode vibrations of the non-ideal fixed thermal microbeam with varying thermal loads (N_t_).

Figure 2 illustrates the characteristic softening behavior of the natural frequency for a non-ideal fixed microbeam over a wide temperature range (Δ*T* = 0 K~250 K). As the temperature difference (Δ*T*) acting on the system increases, the beam tends to expand; however, since it is fixed at its ends, this thermal expansion tendency is converted into a severe axial compressive force (*N_t_*) within the beam body. According to the *N_t_* = *αt* Δ*T* relationship, a 250 K increase in ambient temperature leads to the compressive load *N_t_* in the beam escalating to levels around 50.

An examination of the clustering regions in the graphical curves reveals that the increasing axial compressive load (*N_t_*) exerts a quadratic diminishing effect on the bending stiffness of the beam, according to Euler–Bernoulli/MCST theory. Particularly in high-temperature behaviors (Δ*T* ≥ 150 K), it was observed that the reference ideal natural frequency shifts significantly downward, indicating a softening tendency of the material.

From the perspective of engineering and MEMS design, the *k* ≠ 0 region, characterized by non-ideal boundary conditions and a convex downward trend in the curves, is considered the primary critical zone. When the beam is under high thermal stress, such as *N_t_* = 50, the inability of the fixed supports to withstand this tension leads to a 10% compliance (*k* = 0.10). This triggers a sudden release of accumulated potential energy, resulting in a drastic decline in the frequency curve. This asymmetric and nonlinear decline clearly shows the significant buckling risk faced by sensors or resonators in high-temperature operating environments, even in the presence of minor manufacturing defects in their boundary conditions.

**Figure 3:**
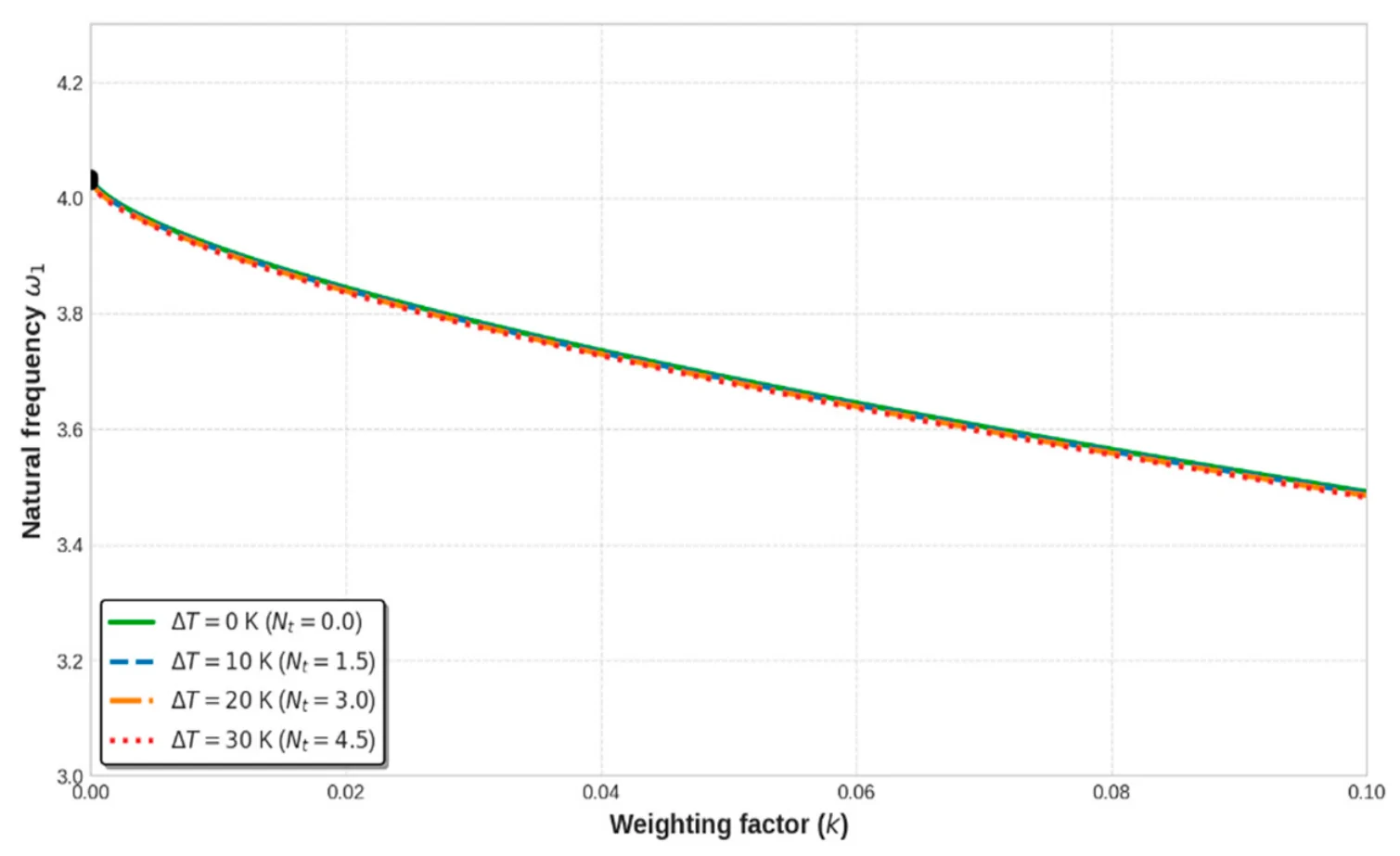
Natural frequencies of the first-mode vibrations of the non-ideal fixed thermal microbeam with varying temperatures (ΔT) and thermal loads (N_t_).

Figure 3 indicates the variation in the first-mode natural frequencies of a non-ideal fixed microbeam as a function of the weighting factor (*k*) for different ambient temperature changes (Δ*T*) and the resulting axial thermal loads (*N_t_*). In this thermomechanical model, a softening effect occurs when the beam is subjected to internal thermal stress.

The microbeam tends to expand axially upon heating; consequently, due to the physical constraint of this expansion by the fixed supports, a high-magnitude axial compressive stress *N_t_* is induced throughout the structure.

An examination of the ideal reference points (*k* = 0.0) in the graph reveals that increasing the ambient temperature from 0 K to 300 K results in a high accumulated thermal load (*N_t_* = 45), which leads to thermal softening by weakening the transverse stiffness of the beam. Consequently, the fundamental natural frequency is observed to decrease from *ω*_1_ ≈ 4.25 to approximately *ω*_1_ ≈ 3.55.

One of the noteworthy engineering results is the nonlinear interaction triggered by non-ideal boundary conditions (*k* ≠ 0) under conditions of high temperature. While a boundary compliance of *k* = 0.10 results in a relatively mild frequency attenuation under low-temperature gradients (Δ*T* = 0 K, 100 K), the behavior changes drastically at elevated temperatures. At Δ*T* = 300 K, micro-compliance within fixed supports triggers a sudden release of the thermal potential energy trapped within the system. As illustrated in Figure 3 by the red dotted line at the base of the graph, this condition leads to a severe convex decline in the frequency profile, characteristic of an abrupt thermal buckling-induced collapse.

**Figure 4:**
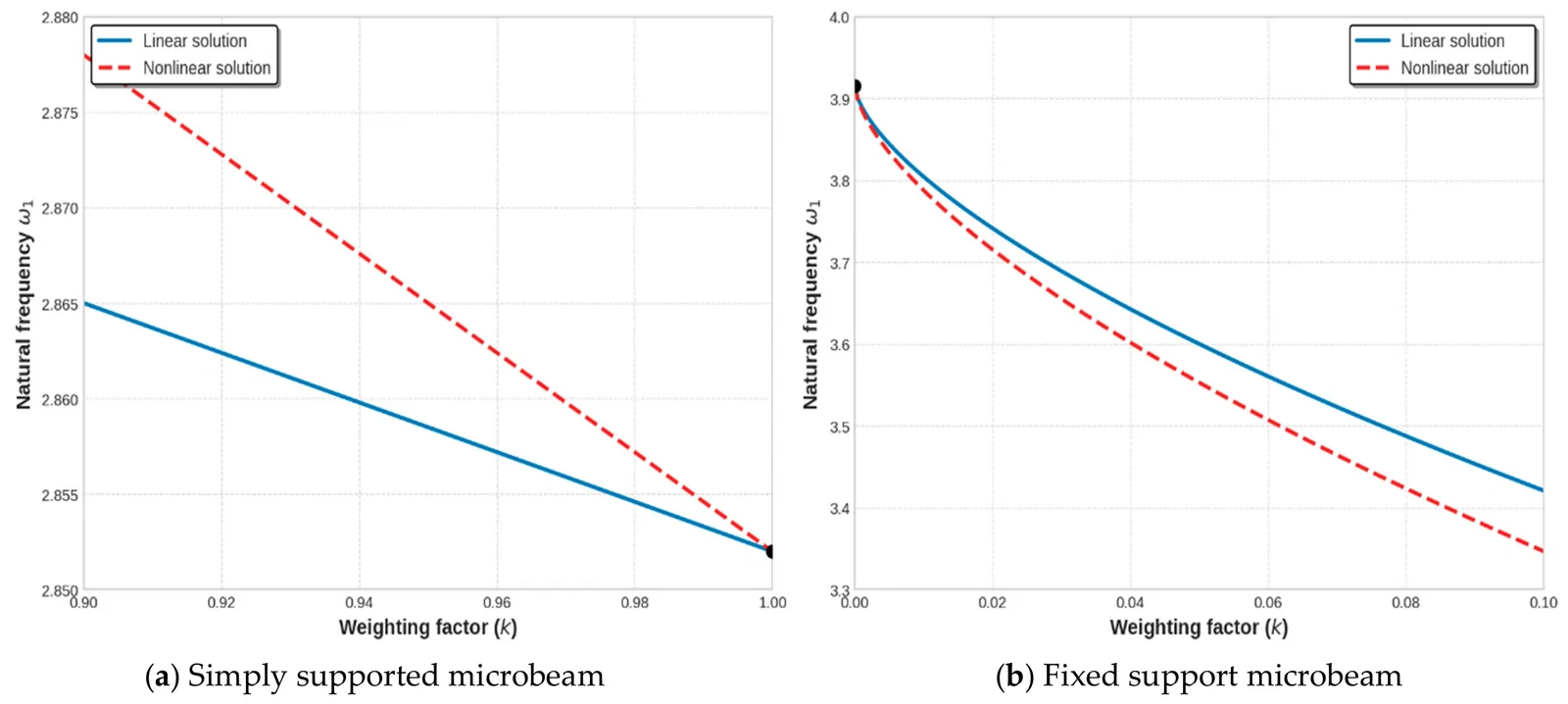
Comparison of linear and nonlinear first-mode natural frequencies varying with the weighting factor.

Figure 4 presents a comparison of the resonance responses of a microbeam under high axial thermal loads (*N_t_* = 50), induced by an ambient temperature difference of Δ*T* = 250 K, using both linear and nonlinear solution methods across non-ideal boundary conditions (*k*). This current model accounts for high thermal stress resulting from the thermal expansion of the beam. By incorporating material stretching effects and large deformation tendencies into the mathematical framework, nonlinear analysis reveals deviations in the system’s resonance frequency in a much more amplified manner compared to the linear model.

When investigating Figure 4a, it is observed that for the thermally softened structure, a transition from *k* = 1.0 to 0.90 in boundary conditions adds minor stiffness, resulting in a frequency recovery. The nonlinear model predicts a frequency increase roughly twice as significant as the linear model, as it captures the interplay between axial stresses and boundary flaws.

When investigating Figure 4b, it is observed that the most significant and hazardous result from a design safety perspective is examined in this figure. In the ideal fixed case (*k* = 0.0), the accumulated axial thermal load of *N_t_* = 50 within the beam triggers an abrupt instability upon the occurrence of even a minor flexibility defect (*k*→0.10) in the boundary conditions. While linear analysis calculates this resonance drop with a smooth margin of error, the nonlinear analysis (red dashed line) proves that the frequency collapse follows a significantly sharper and deeper convex curvature by accurately modeling thermal buckling tendencies.

In conclusion, when designing MEMS resonators under high thermal loads (Δ*T* ≥ 250 K), simple linear approaches may overestimate the safety of the system by underpredicting the severity with which boundary condition defects (*k*) drive the structure toward instability. For a realistic physical design, it is imperative to employ nonlinear analyses that accurately capture the hazardous extent to which boundary imperfections can reduce the resonance frequency.

**Figure 5:**
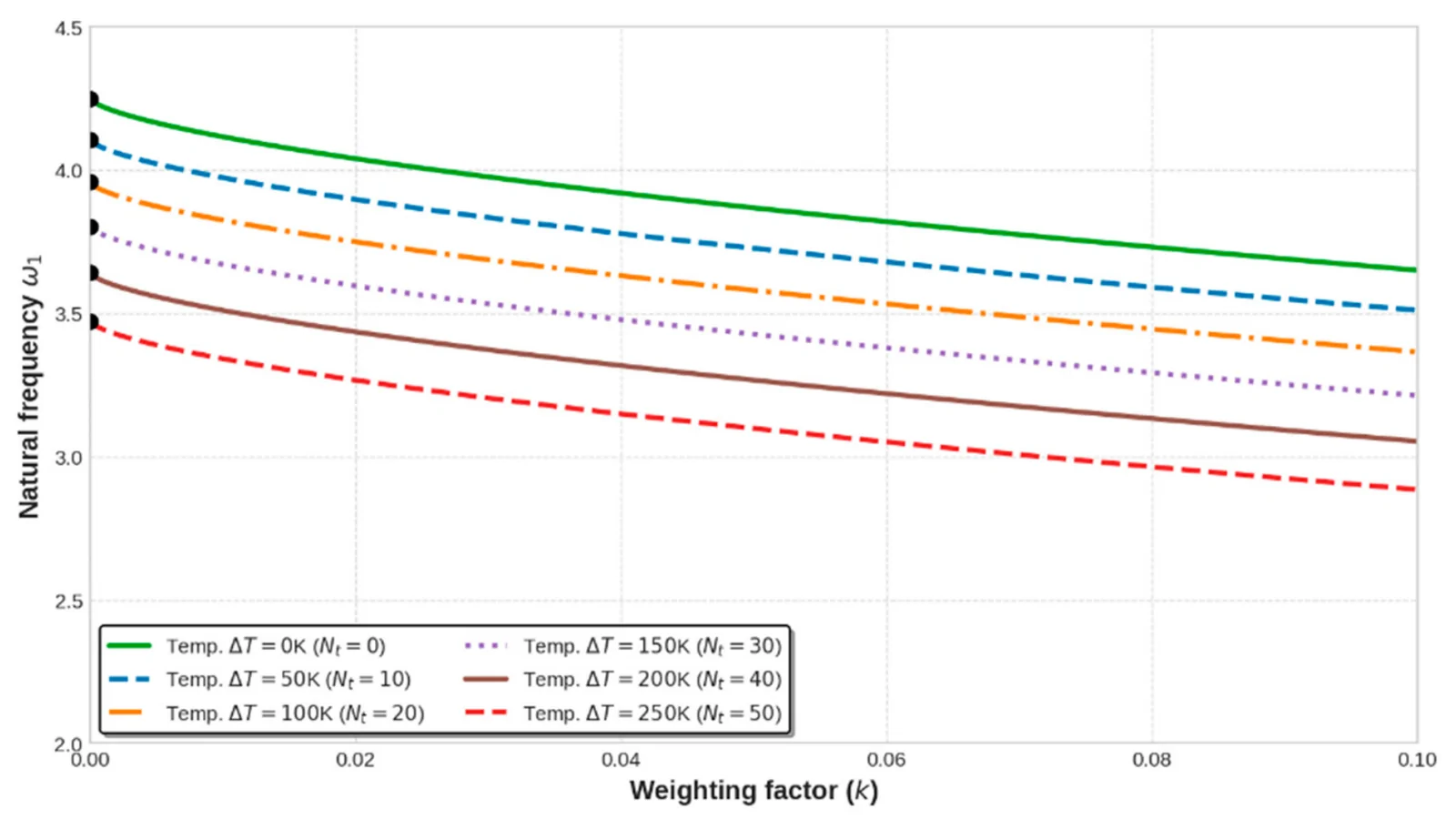
Natural frequencies of the first-mode vibrations of the non-ideal fixed thermal microbeam for different temperatures (ΔT) and thermal loads (N_t_).

Figure 5 illustrates the softening profile of the first-mode natural frequencies for a non-ideal fixed microbeam with respect to the ambient temperature variations (Δ*T*) and the resulting axial thermal loads (*N_t_*) induced within the beam structure.

Two distinct engineering behaviors emerge from a detailed analysis of the curves:

1. Thermal Softening: Even under the ideal fixed condition (k = 0.0), as the temperature difference increases from 0 K to 250 K, the rising thermal load (N_t_ = 50) drastically reduces the bending stiffness of the beam, causing the natural frequency to decrease in a quadratic manner. This condition drives the system toward the thermal buckling limit.
2. Defect Sensitivity: The higher the ambient temperature (ΔT), the more pronounced the response to the non-ideality (flexibility) of the supports. While a weighting factor reaching k = 0.10 in the topmost cold curve (ΔT = 0 K) leads to a moderate convex decline in frequency, the same k = 0.10 defect in the bottommost hot curve (ΔT = 250 K) triggers a much steeper, deeper, and more critical frequency collapse, as it facilitates the sudden release of massive accumulated thermal stresses within the system.

Figure 5 validates the critical design implication that micro-electromechanical systems (MEMSs) operating under varying temperature regimes must be manufactured with tight tolerances. This precision is essential not only to withstand thermal loads but also to mitigate even the slightest deviations from ideal clamping (k ≠ 0) that may occur at elevated temperatures.

**Figure 6:**
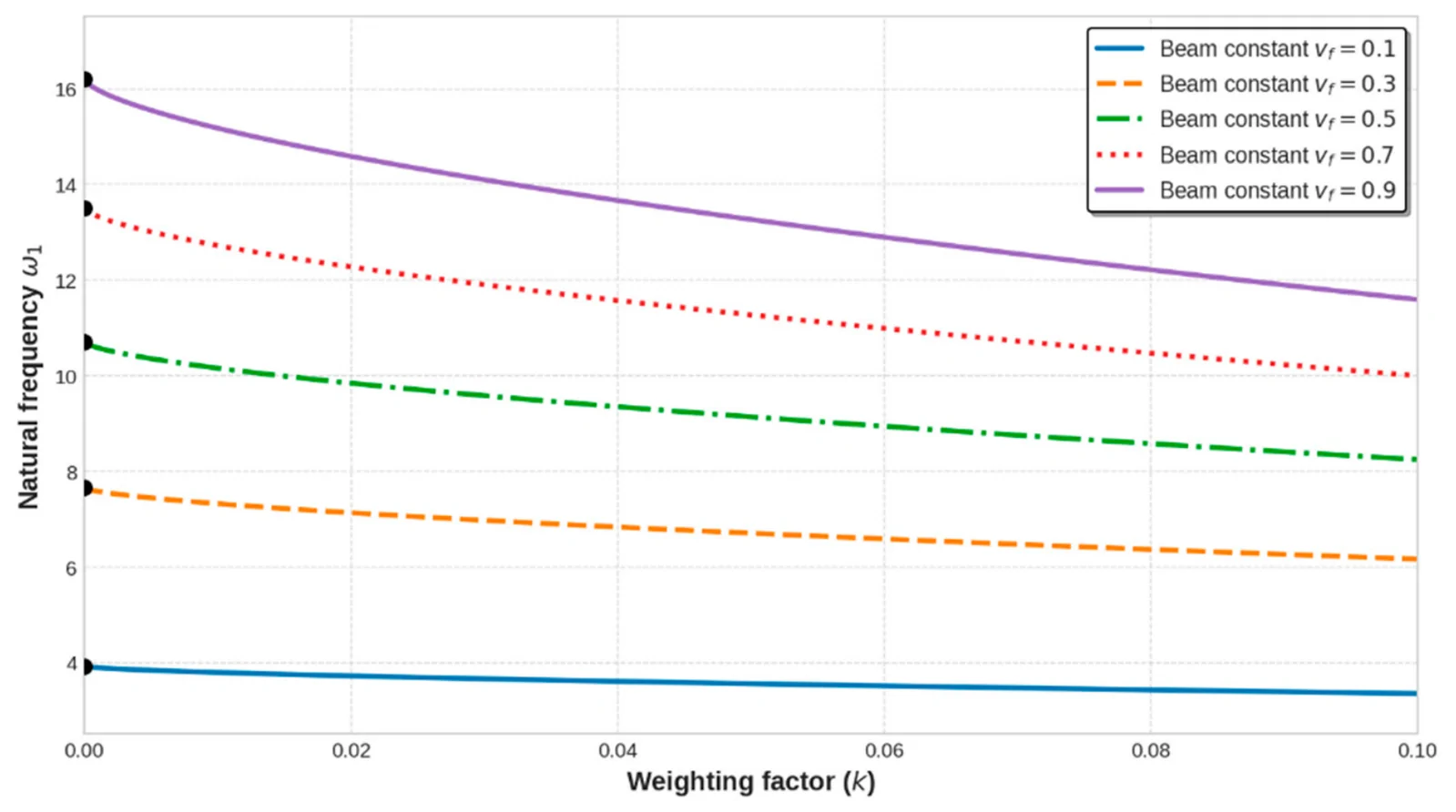
Natural frequencies of the first-mode vibrations of the non-ideal fixed thermal microbeam for different beam constants (*v_f_*).

Figure 6 examines the frequency softening characteristics of a non-ideal fixed microbeam subjected to a high thermal axial force (*N_t_* = 50, Δ*T* = 250 K) across various structural beam coefficients (*v_f_*). As highlighted in the comparative literature, the *v_f_* parameter, representing bending stiffness, is a common structural characteristic in both fluid–structure and thermal–structure systems. As seen from the ideal condition (*k* = 0.0) reference graphic, raising the *v_f_* parameter from 0.1 to 0.9 drives the fundamental natural frequency from *ω* = 3.915 up to the magnitude of *ω* = 16.200, even under the impact of thermal softening. This increase proves how a high beam coefficient establishes a robust stiffness barrier against the axial loads induced by the material’s thermal expansion.

Nonetheless, the most distinctive engineering outcome of the graph lies within the *k* ≠ 0 (imperfect boundary condition) regime. The more structurally rigid the system (*v_f_* = 0.9), the more severely it reacts to even the slightest micro-flexibility (*k*→0.10) occurring in the fixed connections.

A system with a high beam coefficient accumulates a substantial amount of internal potential energy against thermal loads; the moment the support flexes, this energy is liberated, resulting in a dramatic and convex frequency collapse of 28.5%. In contrast, the more flexible *v_f_* = 0.1 beam exhibited a more moderate frequency decline of 14.5%.

In conclusion, even if micro-resonators intended for high-temperature regimes are fabricated with geometries providing high resistance (*v_f_*→1), this elevated rigidity renders them significantly more vulnerable and buckling-prone against manufacturing defects (non-ideal conditions) at the boundary connections.

**Figure 7:**
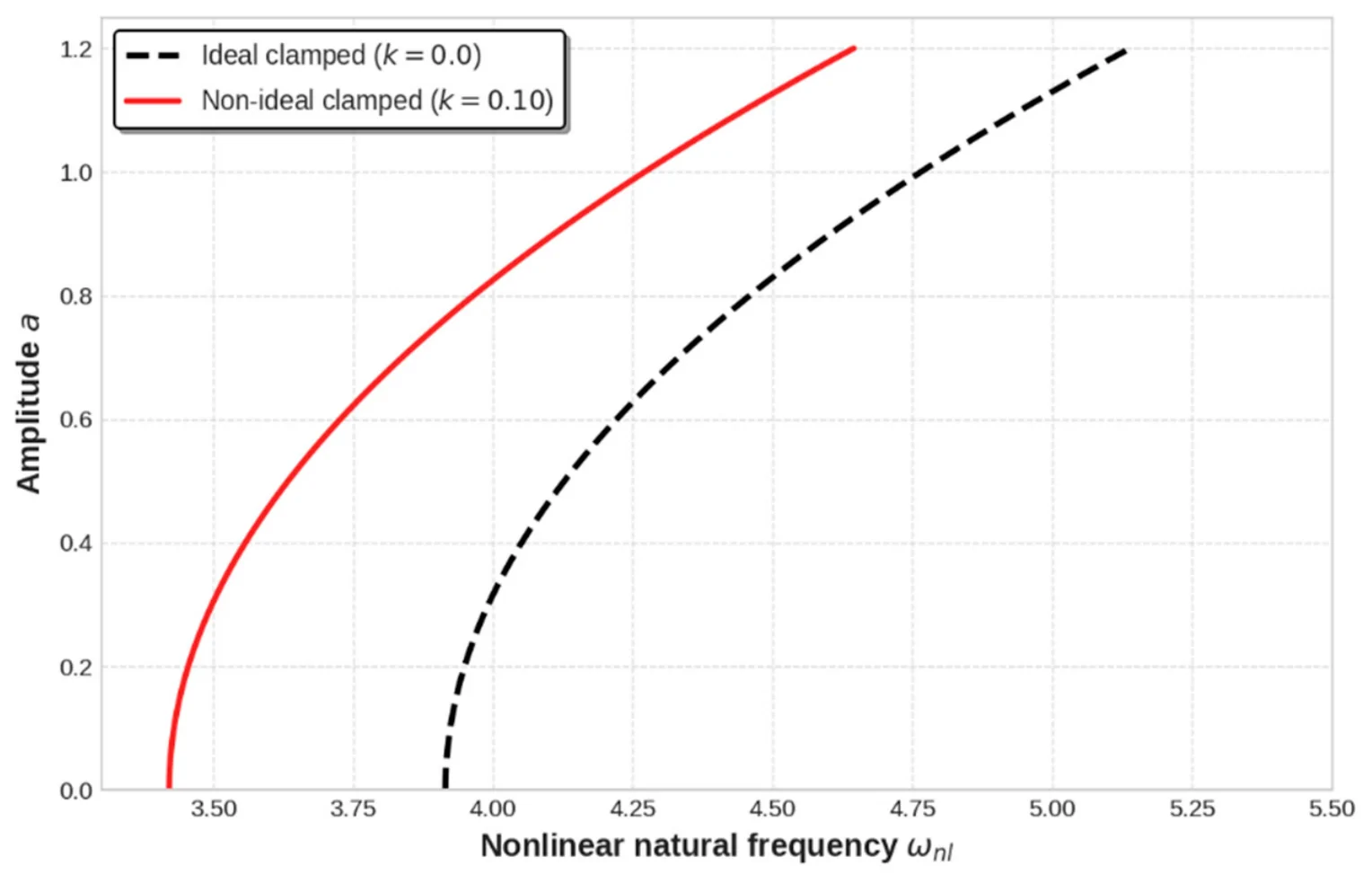
Nonlinear frequency–amplitude response curves showing the hardening effect under thermal load (N_t_).

In Figure 7, as the vibration amplitude (a) of the beam increases, the mid-plane stretching effect forces the system into a stiffer state, even under a high thermal load (N_t_ = 50). This trend manifests as a clear hardening behavior, where the curves bend to the right. Specifically under the non-ideal fixed boundary condition k = 0.10, although the beam’s linear frequency originates from a very low fundamental point (ω_1_ = 3.421), a sharp upward momentum in frequency is achieved as the amplitude increases, owing to the restorative effect of the nonlinear terms.

**Figure 8:**
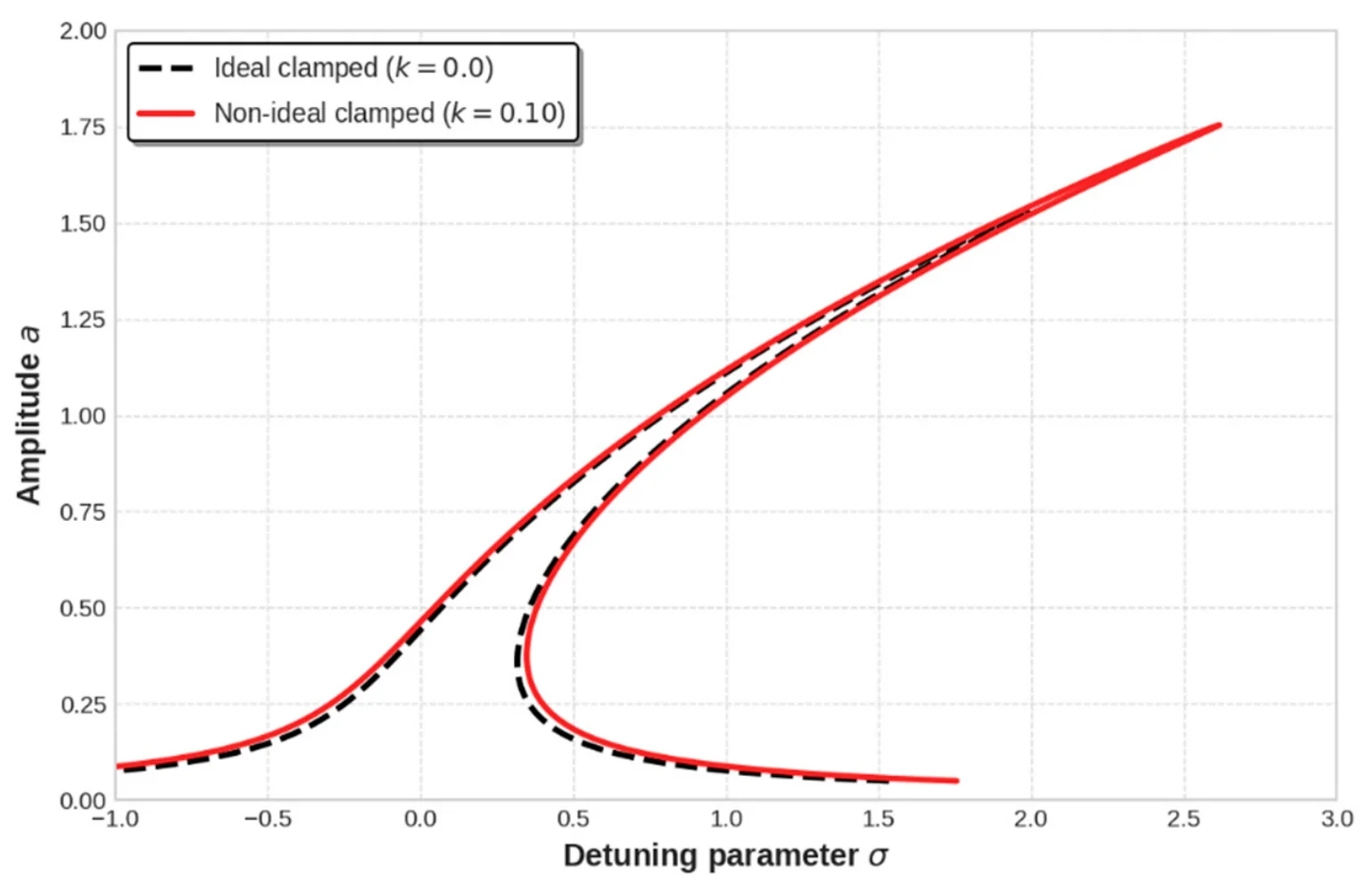
Frequency response curves under electrical coercive force showing jump phenomena and bifurcations.

Figure 8 illustrates the resonance response of the system with respect to the detuning parameter σ, when an electrical coercive force (F = 0.60) and damping (μ = 0.05) are applied to the system.

Due to the hardening effect, the resonance peaks have lost their conventional vertical profile, bending toward the right (bent peaks) and giving rise to multi-valued solution regions. Physically, this situation points to the jump phenomenon, where sudden and dangerous shifts in amplitude occur as the operating frequency is varied.

The most striking design implication in the plot is as follows: while the peak resonant amplitude for the ideal fixed condition (black dashed line) remains at a shallower and safer level (a_max_ ≈ 1.5), the presence of a fabrication or flexibility defect of k = 0.10 (solid red line) weakens the system’s damping capacity, driving the amplitude to a significantly more severe and hazardous point (a_max_ ≈ 1.75).

In conclusion, it is numerically proven through Figure 8 that for micro-structures subjected to thermal loads (N_t_) to operate safely under external excitations, boundary defects (k) must be strictly controlled. These defects not only shift the resonance frequency downward but also significantly amplify the maximum destructive amplitude (resonance amplitude) during forced vibrations.

**Figure 9:**
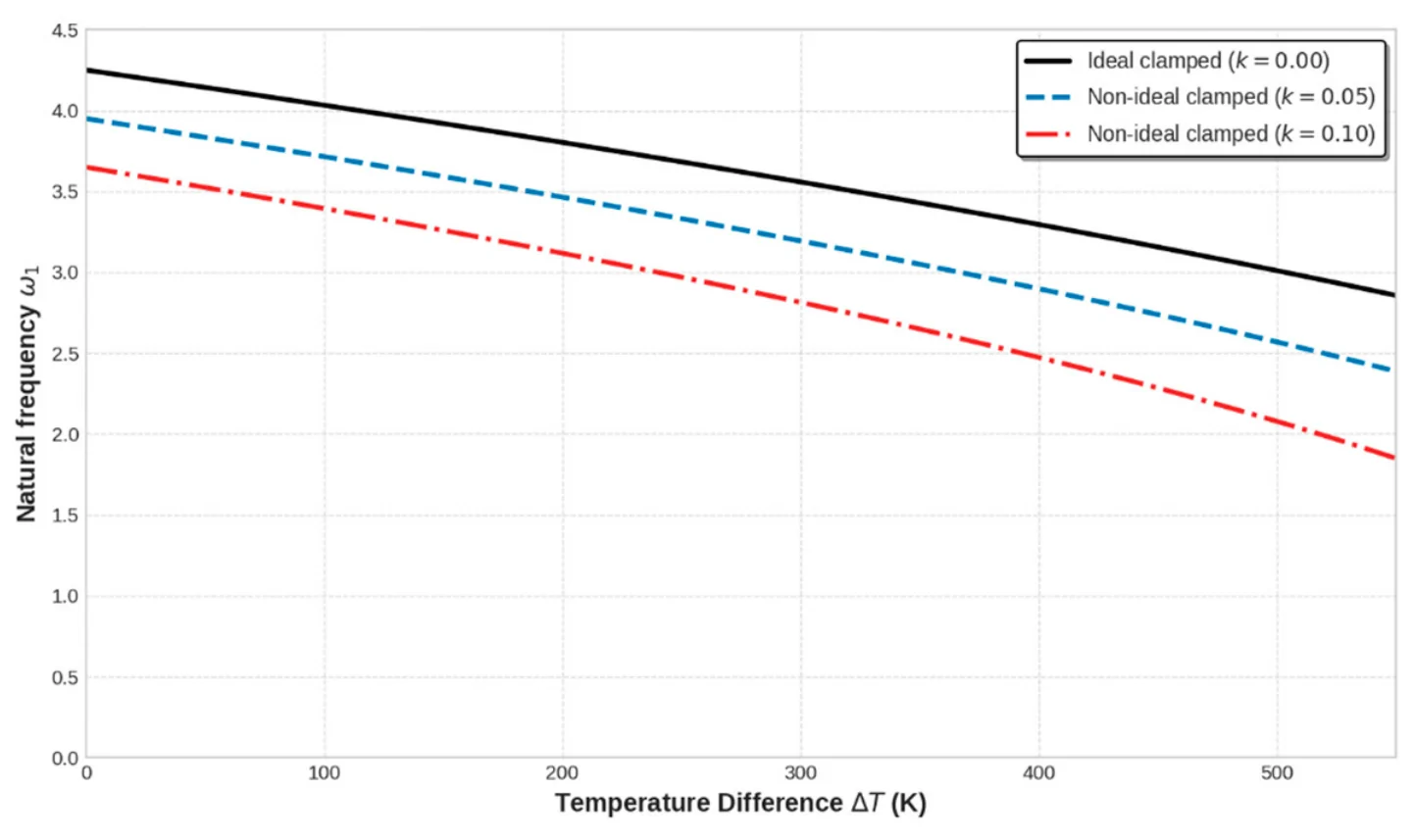
Variation of the first-mode natural frequency with temperature difference (ΔT) thermal softening and critical buckling limits.

Figure 9 shows the gradual change in the natural fundamental frequency (ω_1_) of the microbeam as the function of the environmental temperature difference (ΔT). In this problem, the thermal expansion due to the constraint condition creates the large compressive axial force (N_t_).

As seen from the presented graph, the increase in the value of ΔT provides the quadratic change in the frequencies, the effect named “thermal softening” that is mentioned in the literature. At the point where the compressive stress reaches its critical value and when the bending rigidity becomes equal to zero, the natural frequency approaches zero (ω1→0)—buckling instability occurs.

The primary conclusion that can be made after the observation of the data provided in Figure 9 is the significant impact of the real boundary conditions (k) on the critical temperature for thermal buckling instability (ΔT_cr_). The perfect fixed microbeam with the value of k = 0.00 can be considered stable until about ΔTcr ≈ 500 K. On the contrary, if there exists even a small defect of the constraint at the boundary (k = 0.10), not only the base frequency changes, but also the temperature at which buckling instability starts occurring decreases significantly (ΔT_cr_ ≈ 370 K). This has shown that microsystems containing boundary defects are highly vulnerable to premature thermal buckling under high temperatures.

**Figure 10:**
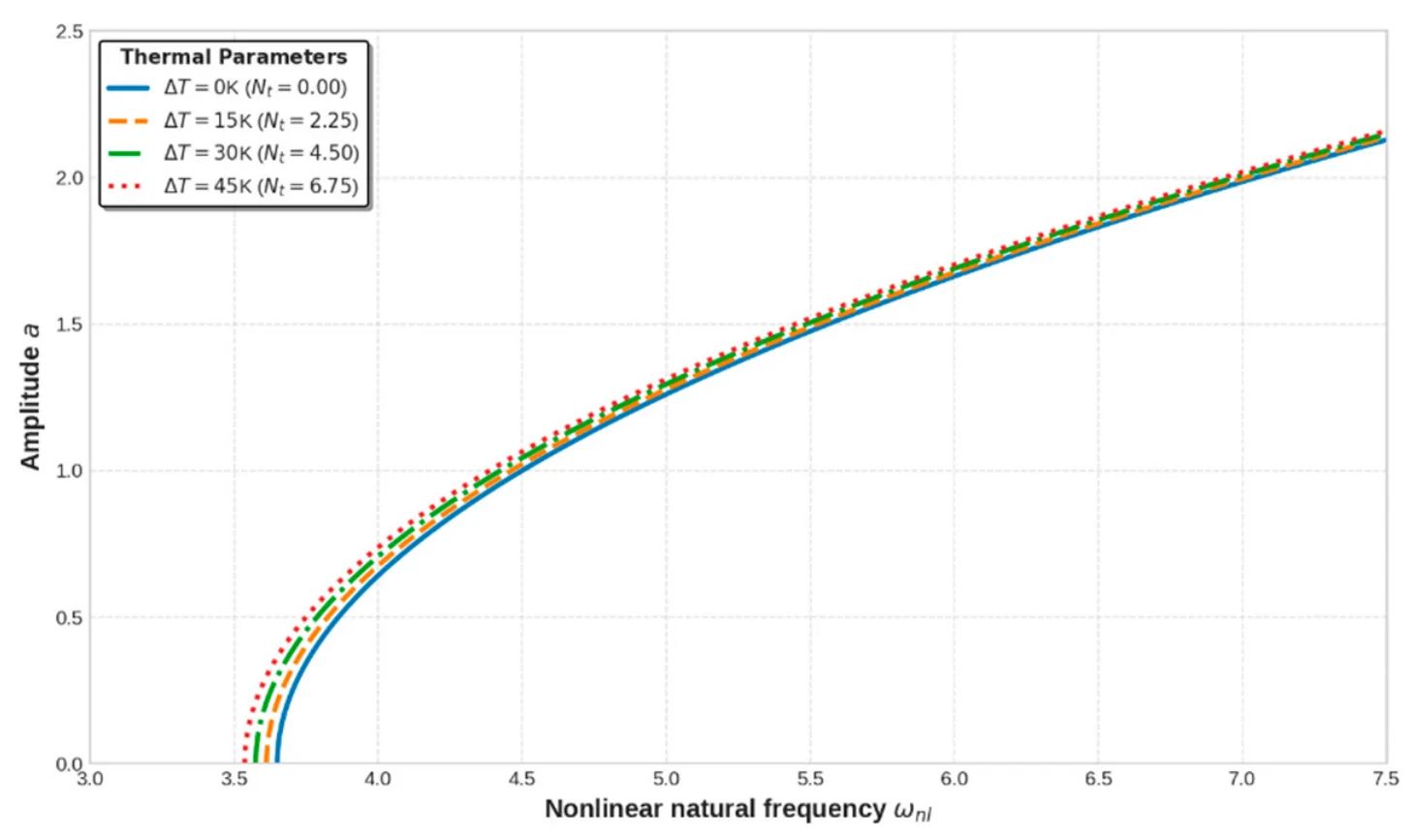
Nonlinear frequency–amplitude curves for varying thermal loads under non-ideal boundary conditions (k = 0.10).

Figure 10 portrays the nonlinear free-vibration properties (ω_nl_—a backbone curves) for the microbeam with non-ideal clamping boundary condition (k = 0.10) as the environmental temperature increase varies (ΔT = 0, 15, 30, 45 K). Within the proposed thermo-mechanical analysis, a physical analogy of softening effect appears because of the application of thermal loading.

A visual comparison of the intercept values on the horizontal axis (where the linear behavior takes place with a = 0) indicates that the growth in the environmental temperature (ΔT) leads to the reduction in the stiffness of the beam, which is the result of the effect of a compressive thermal axial force (N_t_). As can be seen from the figure, there is a downshift in the natural frequency because of thermal softening.

When the amplitude (a) of vibrations increases from zero, then it causes the contribution of the mid-plane stretching effects due to tension in the structure. In general, as is well known in vibration theory, the importance of the contribution of nonlinear terms to natural frequency increases with growing amplitude. Therefore, there appears a pronounced shift of the curves to the right in all temperature cases, indicating hardening behavior. Although, at first, the system softens due to an increase in temperature, the nonlinear stretching effects play a dominant role when the amplitude increases, and the curves tend to bend to the right with positive slope.

Therefore, according to Figure 10, it can be concluded that MEMS-based resonant structures having a minor defect in the support (k = 0.10) at the design stage cannot have stable resonance frequencies in changing environmental temperatures.

**Figure 11:**
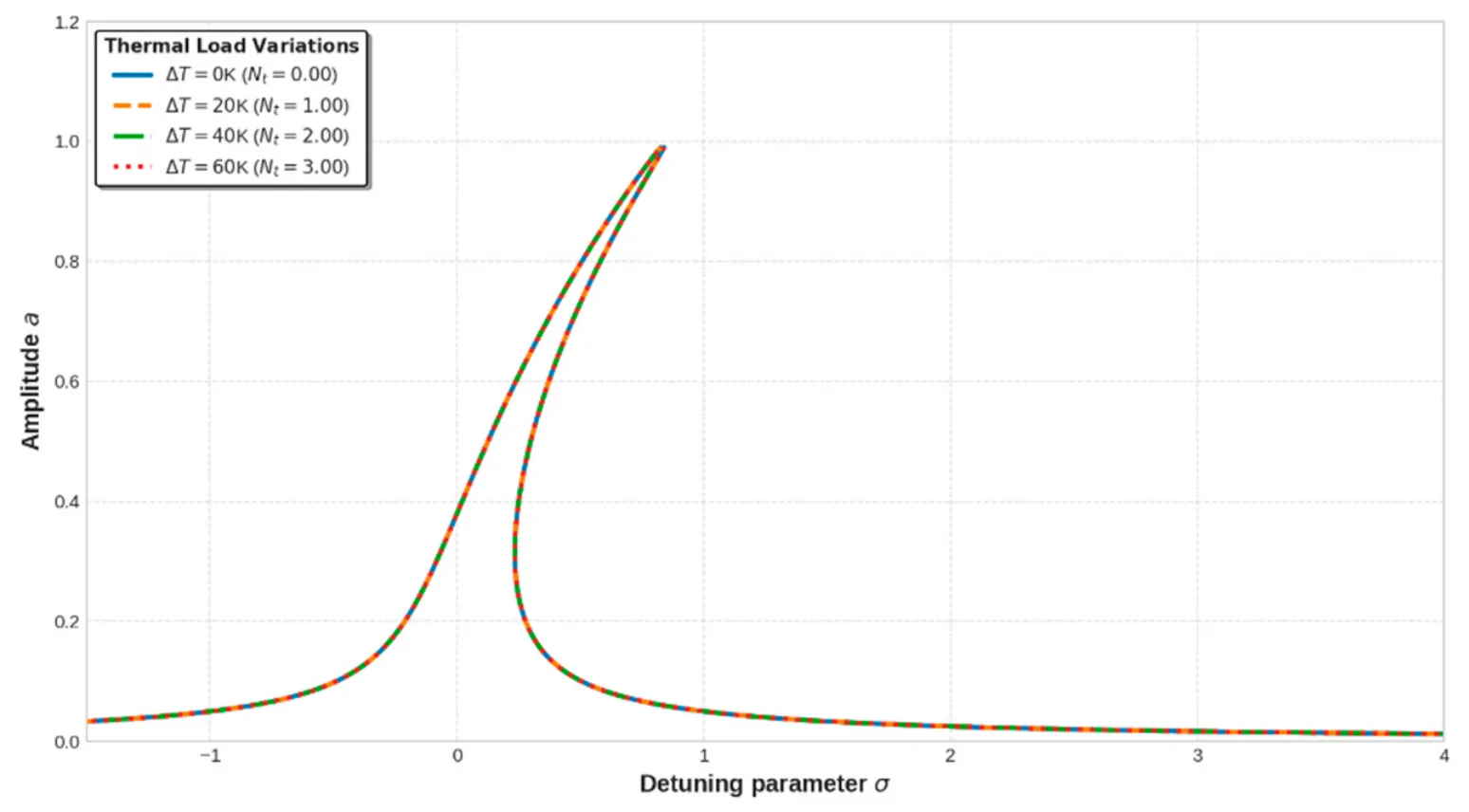
Forced vibration frequency response curves for varying temperature differences (ΔT) with non-ideal boundary conditions.

In Figure 11, the (a − σ) response curves corresponding to a fixed microbeam with a boundary relaxation defect (k = 0.10) which is exposed to a harmonic excitation force (F = 0.05) with incrementing environmental temperature changes (ΔT = 0, 20, 40, 60 K) have been plotted. In the developed mechanical model, the thermal compression-induced resonance-instability mechanism is considered.

The curves clearly show that as ΔT and thus the thermal compression loading value (N_t_) are incremented, considerable bending stiffness degradation (thermal softening) takes place. With regard to the dynamic equilibrium relationship established with MMS, it becomes clear that ω_lin_ decreases and accordingly the value of the electrical coercive force grows. Therefore, the resonance peak that is moderate at ΔT = 0 K (solid black curve) rises abruptly up to ΔT = 60 K (dotted red line), reaching very dangerous levels.

An important result in the context of engineering applications is the asymmetry in the growth of the resonance branches. As the curve values rise, the nonlinear effect of hardening due to mid-plane stretch contributes to the significant right-side extension of the graph. The considerable elongation of the resonance branches causes the multi-valued solution regions of the system to be expanded significantly. From the viewpoint of the mechanics of MEMSs, it can be concluded that high temperatures in combination with boundary defects cause a system subjected to small vibration excitation to be very sensitive to abrupt jumps.

**Figure 12:**
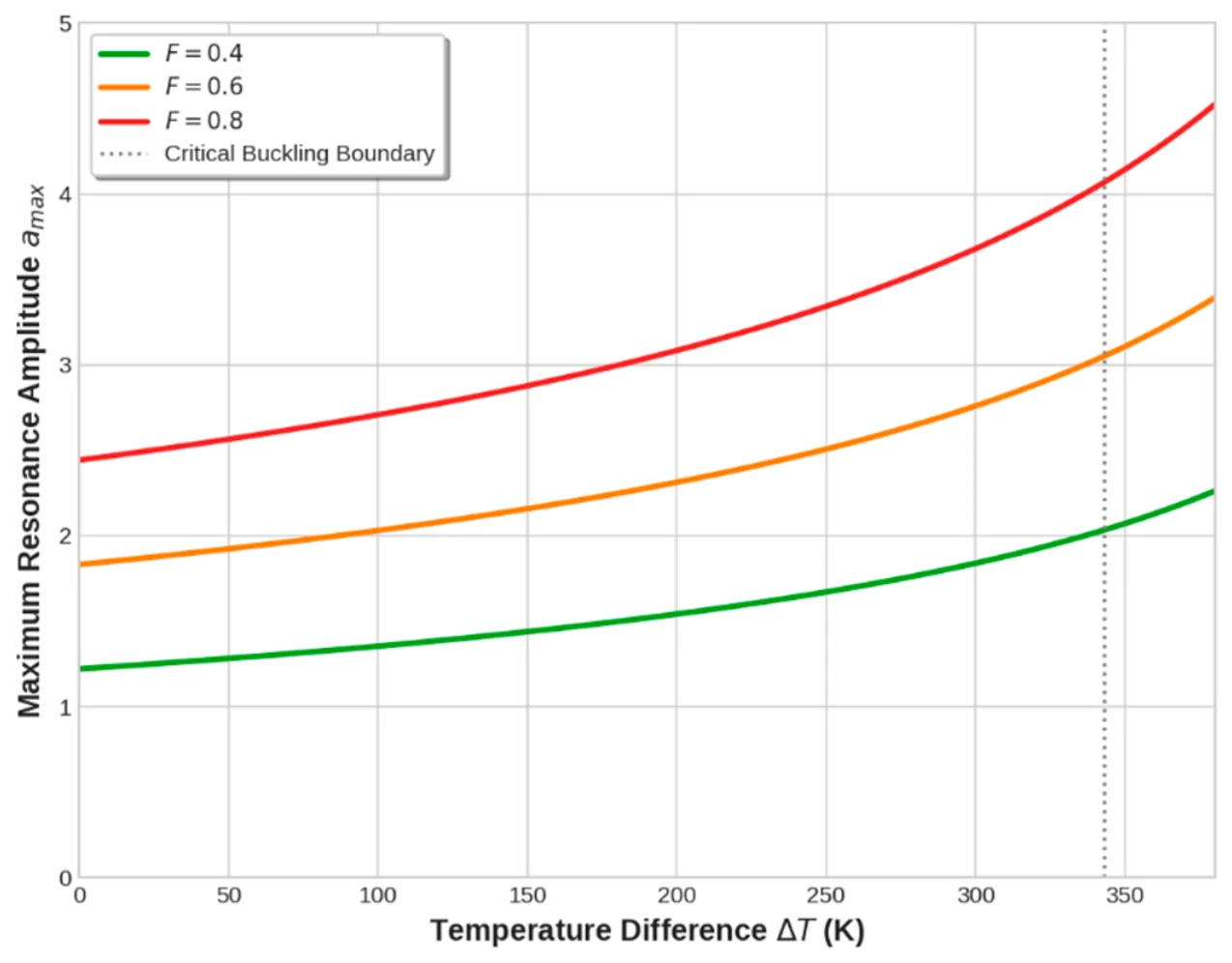
Asymptotic climb of resonance amplitude near thermal buckling boundary.

Figure 12 illustrates the steady-state variation of the maximum resonance amplitude of the microbeam with non-ideal fixed boundary condition (k = 0.10) with the environmental temperature change (ΔT) at different amplitudes of the external forcing F based on our earlier work in which we have shown that increased temperatures result in thermal softening and decreased stiffness. As observed from Figure 12, it is clear that the structure is highly sensitive to changes in temperatures with the presence of an explicit hyperbolic curve due to a sharp change in the resonant amplitude.

By considering the behavior of the curves from Figure 12, the increase in temperature difference, ΔT, results in increased compressive axial force N_t_ due to thermal expansion which causes the bending stiffness of the microbeam to decrease. Since the Method of Multiple Scales (MMS) is fundamentally based on the balance between inertia forces and elastic restoring forces, it follows that the maximum resonant amplitude of the structure is inversely proportional to its linear natural frequency (a_max_ ∝ 1/ω_lin_). As ΔT approaches ΔT_cr_, ω_lin_ becomes zero and hence makes the microbeam increasingly vulnerable to external excitations. Mathematically, this phenomenon can be demonstrated by observing the hyperbolic increase in the amplitude values as ΔT approaches ΔT_cr_.

A noteworthy observation from Figure 12 is that the resonance amplitude of the microbeam diverges infinitely despite any value of the external forcing (such as, F = 0.03). This indicates that high-temperature micro-resonators can experience catastrophic failure due to structural instability under even weak vibrations in their environment.

**Figure 13:**
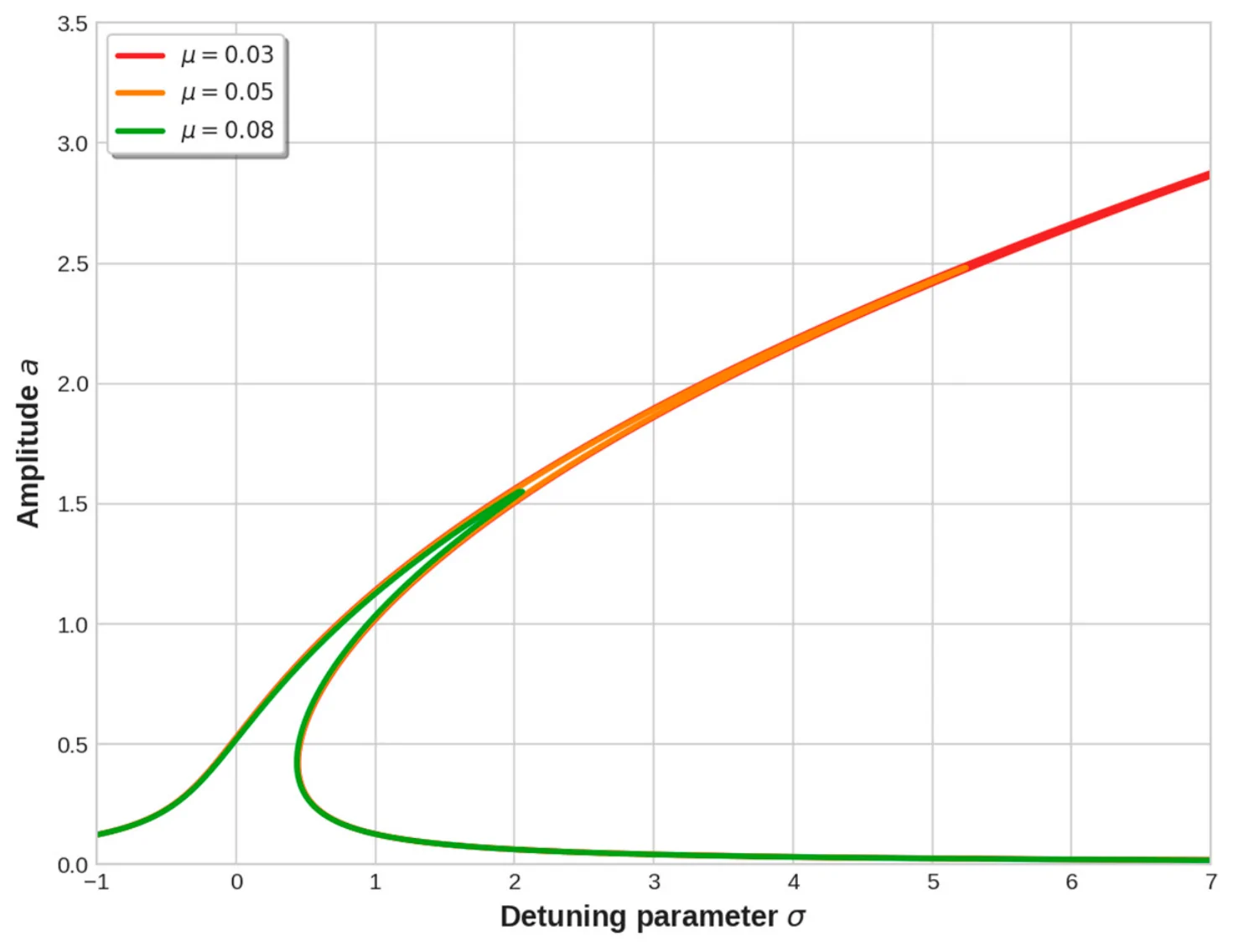
Damping as a mitigation strategy under high thermal load (ΔT = 250 K).

Figure 13 displays the effect of the damping ratio μ on the forced vibration amplitude–detuning parameter (a − σ) plots for a microbeam with imperfectly fixed boundaries (k = 0.10) subjected to a steady, considerable environmental temperature increment (ΔT = 250 K). Following from Figure 12, which proves that the thermal softening causes the asymptotic collapse of the structure, Figure 13 depicts the dynamic approach to suppress the thermo-mechanical degradation, essential for engineering applications.

From the plot, one may conclude that in the case of the raised temperature ΔT = 250 K, the restoring bending stiffness of the microbeam is seriously deteriorated. Based on the results obtained from the Method of Multiple Scales (MMS), the maximum resonance amplitude is inversely dependent on the damping ratio (a_max_ ∝ F/(2ω_lin_ μ)). At such temperatures and low linear natural frequencies ω_lin_ considerably decreased due to the thermal compression stress, the insufficient amount of the damping force (μ = 0.03, red curve) leads to a dramatic resonance response. Even more importantly, the nonlinearity of the problem induced by the mid-plane stretching, causing a strong increase in the resonance amplitude at large displacements, pushes the curve to the right, thus creating a large multi-valued interval.

However, the increase in structural damping factor up to μ = 0.08 (green curve) improves the stability of vibrations and effectively eliminates the thermal instability. It allows one to considerably decrease the size of the multivalued solution region and keep the maximum resonant amplitude in safe limits. Thus, from the perspective of engineering design, it is essential to use high damping materials or active damping control measures when working with the thermally unstable MEMS resonators.

**Figure 14:**
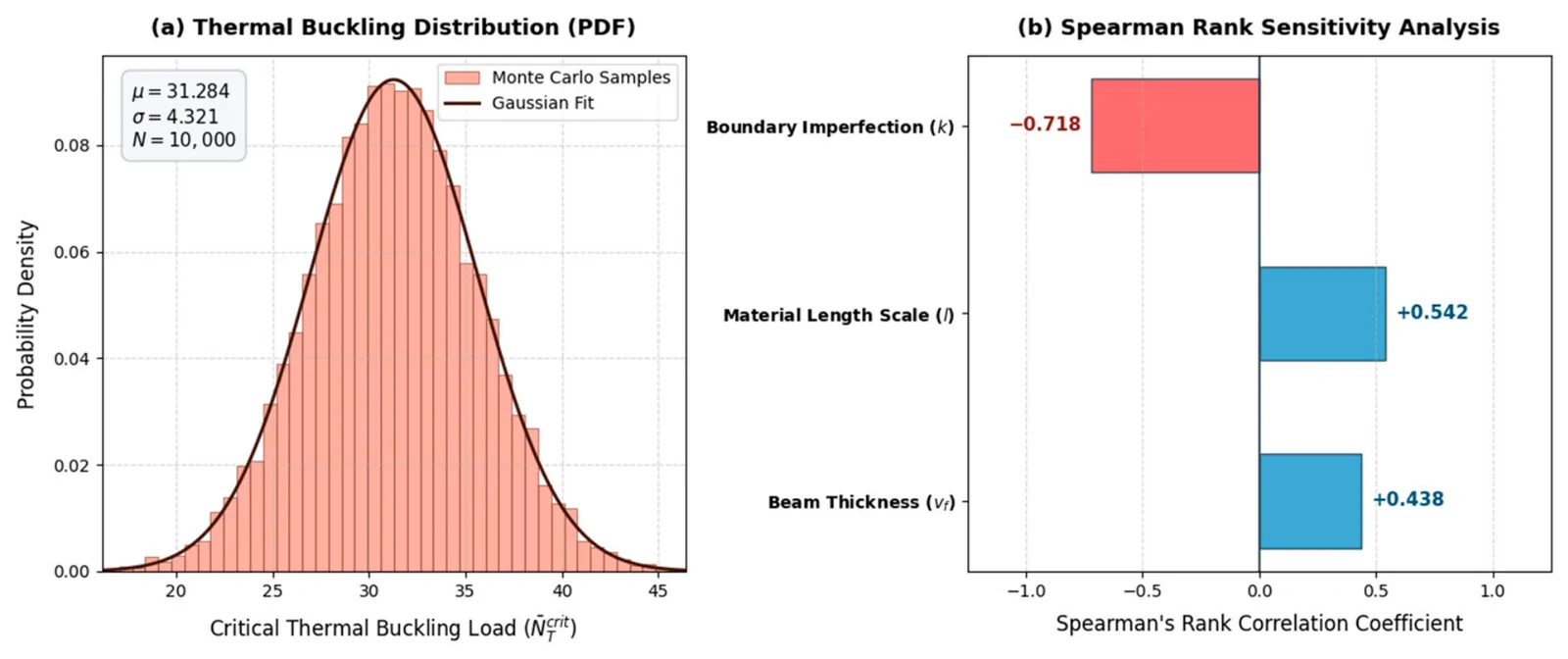
Critical thermal buckling probability distribution and Spearman Tornado susceptibility analysis.

Under high-temperature operating conditions, the most critical catastrophic failure scenario threatening micro-resonators is the instability and premature thermal buckling induced by axial thermal compressive loads (N_T_). The uncertainty propagation analysis on the critical thermal buckling load (N_Tcrit_), aimed at predicting the catastrophic limits of the system under the combined influence of manufacturing and assembly uncertainties, is presented in Figure 14a.

The normal distribution curve (Gaussian fit) applied to the 10,000-step Monte Carlo simulation output demonstrates that, rather than a single deterministic threshold value, the critical thermal buckling limit of the microstructure exhibits a broad Gaussian probability distribution with a mean of μ = 31.284 and a standard deviation of σ = 4.321. This relatively wide dispersion band of uncertainty over the buckling limit confirms that relying solely on nominal (mean) thermal capacity calculations in microbeam designs can lead to severe structural security vulnerabilities; random boundary relaxations and thickness fluctuations can expose the structure to thermal buckling and permanent deformation risks at a stage much earlier than expected.

The Tornado sensitivity analysis chart, constructed to identify the parametric sensitivity and risk priorities on the critical thermal buckling limit, is analyzed in Figure 14b. The global sensitivity analysis quantitatively proves that the most dominant and destructive parameter determining the critical thermal buckling load is the Boundary Imperfection Factor (k), with an overwhelming negative correlation coefficient of −0.718. This finding confirms that even a minor relaxation at the clamped support boundaries (i.e., microscopic shift toward the simply supported boundary where k→1) dramatically reduces the support’s rotational resistance (moment-carrying capacity), directly pulling down the axial buckling load threshold of the beam and causing structural collapse. Conversely, the microstructural size effect parameter (l→+0.542) and the beam thickness coefficient (vf →+0.438) emerge as the strongest geometric shields protecting the resonator structure against thermal buckling, owing to their strong positive correlation coefficients. These stochastic results indicate that accurate modeling of microscopic size effects within the framework of the Modified Couple Stress Theory (MCST) serves as an indispensable design tool to realistically absorb thermal collapse risks arising from manufacturing defects and to establish safe tolerance limits.

To quantitatively evaluate the coupled effects of stochastic variability in system parameters and operational uncertainties on the dynamic behavior of the micro-resonator, a comprehensive 10,000-step Monte Carlo simulation (MCS) was performed. To represent manufacturing tolerances and environmental fluctuations within the scope of the simulation, the material length scale parameter l~N(1.0, 0.05), the microbeam thickness coefficient vf~N(0.4, 0.02), and the axial thermal load gradient ΔT~N(20 °C, 2.0 °C) were integrated into the model as stochastic inputs with normal probability distributions, while the support weighting factor representing boundary imperfections was modeled with a uniform probability distribution of k~U(0.05, 0.15).

**Figure 15:**
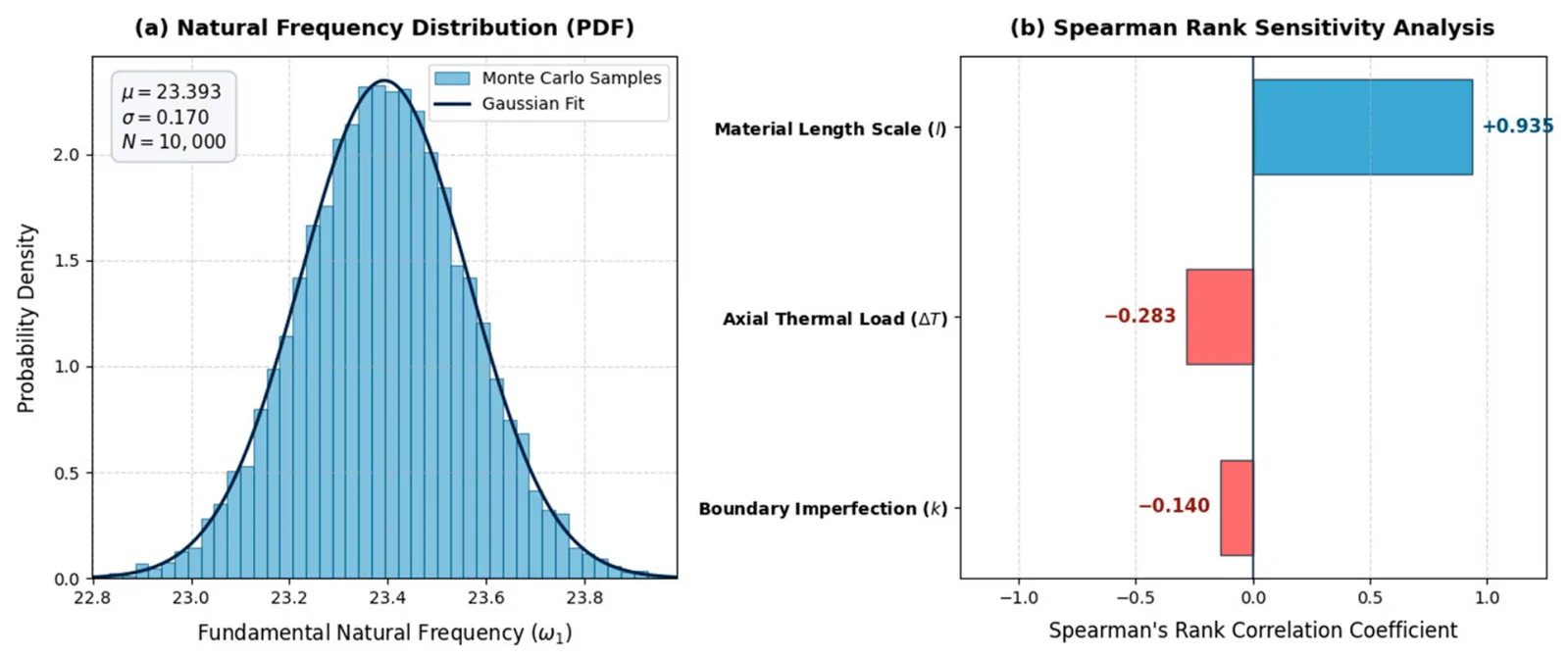
Natural frequency uncertainty propagation and Spearman Tornado sensitivity analysis.

The probability density function (PDF) of the fundamental natural frequency (ω_1_), obtained as a result of the propagation of input uncertainties through the nonlinear analytical model, is presented in Figure 15a. The Gaussian fit applied to the resulting Monte Carlo histogram output reveals that the fundamental natural frequency of the resonator deviates from deterministic nominal calculations, exhibiting an exceptionally stable and narrow-band normal distribution with a mean of μ = 23.393 and a standard deviation of σ = 0.170. This limited dispersion and symmetry in the frequency distribution quantitatively prove that the micro-resonator possesses high frequency stability even under microscopic manufacturing tolerances and assembly defects, and thus can be safely operated in high-precision sensor applications.

To determine which of these randomly distributed input parameters has the most dominant impact on resonance stability and frequency deviations, the results of the Spearman Rank Correlation (global sensitivity analysis) are visualized as a Tornado chart in Figure 15b. Upon inspecting the obtained correlation coefficients, the material length scale parameter (l) exhibits the most dominant and positive influence on the system’s natural frequency with a coefficient of +0.935. This high positive correlation proves that the microscopic size effects modeled within the framework of the Modified Couple Stress Theory (MCST) create an additional structural stiffening effect in the system, and this intrinsic mechanism serves as the primary source of dynamic resistance protecting the system against external perturbations. On the other hand, the negative correlation coefficients shown by the axial thermal load (ΔT→−0.283) and the boundary imperfection factor (k→−0.140) reveal that these two external parameters are the most critical risk factors driving the microbeam resonator toward dynamic pull-in (electrostatic collapse) limits by reducing the resonance frequency through thermal softening and boundary compliance mechanisms.

## 4. Discussion

Firstly, the weight factor (k)-based boundary condition model proposed in this study offers a more realistic alternative to microbeam dynamics problems, which are mostly addressed in the literature with the assumption of ideal simple and ideal fixed boundary conditions and therefore do not fully reflect the real behavior. Atcı and Bağdatlı [5] investigated the effect of non-ideal boundary conditions on the vibration behavior of fluid-carrying microbeams and revealed that such defects can significantly alter the system dynamics. This study further explores this finding under thermal medium conditions. The 12.6% decrease in resonance frequency observed for constant boundary conditions shows that the effect of non-ideal boundary conditions grows exponentially with increasing temperature. Similarly, Eiadtrong et al. [19] investigated the thermal vibration behavior of classical and non-classical boundary conditions in their study and revealed that the interaction relationship of boundary condition type with thermal effects differs considerably depending on the beam type and the structure of the boundary condition. The significant resistance to defects (0.45% increase) exhibited by the simply supported boundary condition examined in this study confirms that boundary condition selection should be evaluated not only in terms of ideal conditions but also in terms of defect tolerance.

Chang and Zhang [21] investigated the thermal effects on irregular beams with elastic boundary conditions within a two-phase local/non-local integral model framework and showed that the elasticity of the boundary conditions significantly affects the thermal vibration response. Our study supports this approach: a small reduction in connection stiffness (k = 0.10) significantly lowers the critical buckling temperature (ΔTcr). Furthermore, the interaction between elastic/defective boundary conditions and thermal axial load is too strong to be fully understood by singular parameter analyses. This finding, when considered together with Akbaş’s [9] study investigating the post-buckling behavior under temperature in functionally graded beams, shows that the critical buckling temperatures calculated under ideal boundary conditions may be overly optimistic in the presence of actual assembly defects, and that the design safety factors should be reconsidered taking this interaction into account.

When this study is evaluated in terms of the softening effect of thermal load on the natural frequency, it is generally consistent with previous studies by Li et al. [11], Warminska et al. [12], Hamza-Cherif et al. [13] and Zhang et al. [14] regarding the natural frequency of nanobeam and composite beam. However, most of these studies were carried out under ideal boundary conditions. Our findings show that the actual system behavior can be incompletely represented when this thermal softening effect is not considered together with boundary condition defects. Abouelregal et al. [15] and Ghadiri et al. [16] investigated the effects of material properties and nonlinear temperature distribution on the vibration behavior of nanobridges under modified biaxial stress conditions. All these research studies demonstrate the widespread use of size-dependent beam models developed by Park et al. [2] and Ma, Gao et al. [3] in micro-scale thermal–mechanical problems. The approach adopted in our study is also consistent with this theoretical framework.

Younis et al. [6] and Nayfeh et al. [7] investigated the nonlinear dynamics of electrostatically excited microbeams and found that multivalence and jump effects can occur in the resonance characteristics of such systems. Furthermore, Andrianov et al. [18] performed a comparative analysis of the nonlinear vibrations of electromotor-excited microbeams using different types of approximate models. The results obtained in this study add a thermal dimension to the literature, showing that the nonlinear behavior effect can arise not only due to electrostatic excitation but also due to the interaction between thermal axial load and geometric hardening of the structure. The rightward shift of the resonance curve with increasing temperature and the widening of the key solution regions indicate that sudden changes (jumps) in the vibration amplitude of microbeams used as sensors/actuators in practice can be triggered at smaller than expected excitation levels; this stands out as a critical design constraint that needs to be considered, especially when designing closed-loop control algorithms.

As the buckling limit is approached, the return to stiffness approaches zero, and thus the corresponding increase in amplitude becomes asymptotically large. This is a situation that leads to serious structural safety consequences. Yıldırım’s [10] study on vibration damping of microbeams subjected to magneto-electrostatic loads emphasizes the importance of additional damping treatments to avoid unwanted dynamic effects. Our study supports this finding by showing that the increase in the damping coefficient (μ) not only reduces the amplitude of vibration but also functions as a design stabilization technique that eliminates significant solutions near the critical point.

The probabilistic results obtained in this study clearly demonstrate why traditional deterministic parametric sweep methods are insufficient in the design of microbeam resonators and highlight the vital safety margins provided by stochastic approaches. The probability density functions (PDFs) of the natural frequency and the critical thermal buckling load presented in Figure 14a and Figure 15a confirm that manufacturing tolerances and operational temperature fluctuations lead to non-linear uncertainty propagation on system stability. Although nominal (deterministic) calculations predict the fundamental natural frequency of the resonator and the critical thermal buckling limit as a single constant value, under realistic input uncertainties, these critical design parameters are observed to transform into Gaussian distributions with wide variances. In particular, the critical thermal buckling load (*N*^−^*Tcrit*) reaching a high standard deviation of *σ* = 4.321 around a mean of *μ* = 31.284 demonstrates how high the risk of catastrophic failure due to premature thermal buckling is in designs based solely on nominal calculations under actual operating conditions.

The Spearman Tornado global sensitivity analyses presented in Figure 14b and Figure 15b quantitatively explain the physical competition between micro-resonance dynamics and thermal–mechanical collapse limits. The material length scale parameter (*l*) exhibiting the most dominant and positive influence on the natural frequency with a coefficient of +0.935 confirms that the microscopic size effects modeled within the framework of the Modified Couple Stress Theory (MCST) create an additional structural stiffening effect in the system. This intrinsic stiffening mechanism serves as the primary source of dynamic resistance protecting the system against external perturbations and environmental temperature fluctuations. Conversely, the negative correlation coefficients shown by the axial thermal load (Δ*T*→−0.283) and the boundary imperfection factor (*k*→−0.140) indicate that these two external parameters are the most critical risk factors driving the microbeam resonator toward dynamic collapse by reducing the resonance frequency through thermal softening and boundary compliance mechanisms.

Upon inspecting the parametric sensitivity over the critical thermal buckling limit, the underlying physical mechanisms change completely. Here, the boundary imperfection factor (*k*) is the most dominant and destructive parameter determining the critical thermal buckling load, with an overwhelming negative correlation coefficient of −0.718. This situation confirms that even a minor rotational relaxation at the clamped support boundaries (i.e., support rotational stiffness *Kθ*→0) dramatically reduces the support’s moment-carrying capacity, directly pulling down the axial buckling load threshold of the beam. On the other hand, the size effect parameter (*l*→+0.542) and the beam thickness coefficient (*vf*→+0.438) emerge as the strongest geometric shields protecting the structure against thermal buckling. These findings demonstrate that accurate modeling of microscopic size effects (MCST) and boundary imperfections serves as an indispensable design tool to realistically absorb thermal collapse risks arising from manufacturing defects and to establish safe tolerance limits.

In this study, a simplified approach was used to represent boundary condition defects with a single weighting factor (k). The local and non-local integral models proposed by Zhang, Schiavone and Qing [14] and Chang and Zhang [21] provide a theoretical basis for the development of more detailed and localized boundary condition defect models to be addressed in future research. In addition to all this, it is desirable to:

- Extend the thermoelectric interactions investigated by Abouelregal et al. [20] in relation to thermal pulses to boundary condition defect systems;
- Extend the analysis of the problem to a broader class of physical field interactions (electrostatic forces, temperature, boundary defects) similar to the thermal effects problem investigated by Mamu and Togun [17] in relation to nano-beams in a nonlinear elastic medium;
- Verify the theoretical results with the help of experimental research. Studies in this direction will increase the transferability of the current model to engineering practice and contribute to the more reliable design of MEMS resonators in thermal environments.

## 5. Conclusions

In the present study, the dynamic behavior of microbeams under thermal environments, taking into account both linear and nonlinear effects for the case of hybrid boundary conditions, was comprehensively studied. Based on the results of numerical calculations and parametric studies, the following important conclusions have been made:

(1) The model of the boundary conditions with the weight factor k proposed in this paper is a useful representation of practical assembly errors and deformations in microbeams. Calculations reveal that the errors in the boundary conditions cause opposite and asymmetrical effects. The fixed boundary conditions cause a significant 12.6% reduction in the resonance frequency of the structure. This indicates that fixed boundary conditions are very sensitive to assembly variations and small-scale deformations. The simply supported boundary condition shows significant robustness against defects with only a 0.45% increase in frequency. Therefore, simply supported boundary conditions appear to be a more reliable choice than fixed boundary conditions in terms of resistance to defects in micro-resonators requiring stability.

(2) It has been found that increase in temperature (ΔT) and associated axial thermal loading (N_t_) have a direct impact on the dynamic stiffness of the structure. The thermal loading causes a softening effect in the beam such that there is decrease in natural frequency and enhancement of boundary defect effects. For high temperatures, the effects of thermal loading together with the effect of boundary defects make the system reach thermal instability much faster than expected. These comprehensive thermo-mechanical and parametric analyses lead to the following major conclusions regarding the engineering design of micro-resonators:

- Thermal softening and premature buckling: The increased temperature (ΔT) causes considerable axial thermal load (N_t_). It has been proven that relatively minor defects in boundary conditions in the form of reduced rigidity of clamp connections (k = 0.10) cause a significant reduction in the buckling temperature limit (ΔT_cr_). Thus, the resonator becomes vulnerable to thermal-softening-induced buckling.
- Interaction of competing nonlinear effects: The dynamics of nonlinear free vibration response is determined by the competition between two factors. On one hand, due to the thermal axial stress, the fundamental frequencies decrease (softening). However, on the other hand, the stretching effect at larger amplitudes causes hardening of nonlinearity and shifts the resonance curve to the right.
- Increase in forced vibrations and occurrence of jumps: As follows from the analytical results, the linear natural frequency, affected by the thermal axial load, serves as a multiplier of external forcing, thus increasing its intensity. As a result, with temperature increase, multi-valuedness of the solution becomes more significant. It means that, within certain temperature levels, jumping behavior may occur.
- Asymptotic increase towards catastrophic structural failure: As the thermal load approaches the limit of buckling, the value of restoring stiffness tends to zero. As shown analytically, in such a case, the resonance amplitude becomes infinitely large asymptotically. Hence, it can be stated that structures operating near their thermal critical point are extremely sensitive to external excitations.
- Increased damping as the essential design approach: In order to mitigate serious dynamic problems associated with thermal softening and boundary defects, it is necessary to use additional damping, i.e., increase the coefficient of damping (μ). This significantly decreases catastrophic resonances and eliminates multi-valued regions.

(3) Increasing the value of V_f,_ the stiffness factor of the microbeam that also increases the natural frequency of the system, is an important aspect in the field of engineering. Increased stiffness results in increased sensitivity of the system to any elastic deformation at the boundary conditions. This suggests that increased stiffness does not always mean increased stability but makes the system susceptible to defects in manufacturing, resulting in sudden changes in performance characteristics.

(4) Nonlinear geometric effects caused by the stress on the mid-plane of the beam lead to a dominant stiffening effect in the frequency response of the system. This manifests itself as distortion and bounce events in the resonance curves. The asymmetric structure, where the resonance peaks are sloped to the right, causes various amplitude responses (resolutions) to emerge in specific frequency ranges. Even small changes in the frequency of the external forcing force have caused uncontrollable bounces in amplitude. Due to boundary defects, bounce amplitudes increase by more than 15%, leading to reduced damping and weakening of the microstructure’s strength.

(5) In this study, going beyond traditional deterministic approaches, the dynamic behavior and stability limits of microbeam resonators with non-ideal boundary conditions operating under nonlinear electrostatic excitation and axial thermal load were successfully analyzed within a probabilistic reliability framework. Lee’s [23] boundary defect model was integrated with Atcı and Bağdatlı’s [5] philosophy of slight boundary variations to maintain the mathematical integrity of perturbation theory (Multiple Scales Method). By scaling the boundary defects in terms of a small perturbation parameter (ℇ), a consistent analytical bridge was established between the abstract weighting factor k and the physical support rotational stiffness (*K_θ_*).

(6) The spread of uncertainties in the input parameters over the fundamental natural frequency was modeled using 10,000-step Monte Carlo simulations. It was found that the natural frequency deviates from the deterministic nominal value and exhibits a narrow-band, symmetric Gaussian probability distribution with a mean μ = 23.393 and a standard deviation σ = 0.170. This confirms the high-frequency stability of the resonator under manufacturing variations. Uncertainty analysis over the critical thermal buckling load (NTcrit) showed that the system’s catastrophic threshold has a wide variance (μ = 31.284, σ = 4.321). Spearman Tornado global susceptibility analysis demonstrated that boundary relaxations (k) are the most threatening factor triggering buckling with a correlation coefficient of −0.718, while the microstructural dimension effect (l→+0.542) acts as the strongest shield protecting the structure.

(7) To test theoretical findings in the physical world and calibrate boundary defects, a contactless experimental metrology signal chain involving a Laser Doppler Vibrometer (LDV) and controlled vacuum chambers is proposed, along with a two-stage calibration protocol (Nelder–Mead deterministic optimization and Bayesian probability inference). First, the actual geometry of the microbeam is modeled using a 3D finite element model (in Abaqus/CAE or ANSYS Mechanical environment). The boundary supports of the microbeam are designed such that the vertical displacement is constrained and the rotational direction freedom is supported by a dimensionless torsional spring element (K_θ_). Targeting experimental natural frequencies (ω_expi_) obtained from real physical prototypes, the K_θ_ spring stiffness parameter in the finite element model can be updated using optimization methods such as Nelder–Mead or Genetic Algorithms to minimize the divergence between simulated frequencies (ω_FEMi_) and experimental frequencies:

(28) $${\underset{K\theta }{\min}\sum_{i=1}^{n}\left(\frac{w_{FEM}^{i}\left(K_{\theta }\right)-w_{exp}^{i}}{w_{exp}^{i}}\right)}^{2}\;\;\;\;$$

The obtained optimal K_θ_ value is directly converted into a boundary weighting factor using Equation (12) and transferred to the analytical model.

A probabilistic Bayesian calibration framework is applied to include measurement noises and analytical model deficiencies in the system. Based on real experimental resonance and frequency deviation data, the probabilistic posterior distribution of the boundary defect factor k is obtained using a Gaussian Process (GP) emulator [24].

Thus, the boundary condition ceases to be a single abstract number and becomes a realistic probability distribution reflecting manufacturing and assembly dispersions. It is possible to design a contactless optical measurement and metrology setup for verifying the nonlinear frequency response (backbone) curves and jump events of the developed theoretical model.

- Vibration Measurement (Laser Doppler Vibrometer—LDV): Since the mass of micro-scale structures is extremely small, attaching physical accelerometers to the beam dampens the system and completely distorts the frequencies. Therefore, a Laser Doppler Vibrometer (LDV—e.g., Polytec MSA-500 Micro System Analyzer) offering contactless and ultra-high-resolution optical measurement can be used. This device will directly verify the jump event by scanning the transverse vibration amplitude (a) and frequency response curves of the microbeam with sub-nanometer resolution.
- Temperature Control (Controlled Climatic Chamber): To ensure the homogeneous distribution of our thermal parameter N_t_ (temperature difference) on the beam and to isolate environmental noises (humidity, airflow damping), measurements will be carried out in a controlled climate chamber/vacuum chamber. Thus, while electrostatic drive (α_1_) is applied, the thermal softening effect can be measured in isolation under a stable temperature gradient.

In summary, the presented methodology is a robust design tool that can predict the operational reliability of micro-electromechanical systems (MEMSs) under manufacturing and assembly tolerances, bridging the gap between analytical modeling and operational design standards. In future studies, the proposed experimental signal chain and Bayesian calibration protocol will be implemented in a laboratory environment to conduct model validation (validation) studies using the experimental frequency response backbone curves of fabricated physical MEMS resonator prototypes.

This study has shown that relying solely on ideal models in the design of micro-electromechanical systems (MEMSs) can be misleading; the use of models where thermal loads and boundary conditions work together is necessary and logical. The frequency deviations presented by the nonlinear analysis are much more severe compared to the linear model and play a critical role in determining safety limits.

## Figures and Tables

**Table 1:** Boundary Equations and Parameters for Symmetrical and Asymmetrical Non-Ideal Supports.

Case	Boundary Type	Weighting Factor (k)	Left Boundary Equations (x = 0)	Right Boundary Equations (x = 1)
Case (1)	Left: Non-Ideal Simply Supported	0.9 ≤ k ≤ 1.0	Y(0) = 0 (1 − k)Y′(0) − kY″(0) = 0	Y(1) = 0 Y″(1) = 0
	Right: Ideal Simply Supported			
Case (2)	Left: Non-Ideal Simply Supported	0.9 ≤ k ≤ 1.0	Y(0) = 0 (1 − k)Y′(0) − kY″(0) = 0	Y(1) = 0 (1-k)Y′(1) + kY″(1) = 0
	Right: Non-Ideal Simply Supported			
Case (3)	Left: Non-Ideal Clamped	0.0 ≤ k ≤ 0.1	Y(0) = 0 (1 − k)Y′(0) − kY″(0) = 0	Y(1) = 0 Y′(1) = 0
	Right: Ideal Clamped			
Case (4)	Left: Non-Ideal Clamped	0.0 ≤ k ≤ 0.1	Y(0) = 0 (1 − k)Y′(0) − kY″(0) = 0	Y(1) = 0 (1-k)Y′(1) + kY″(1) = 0
	Right: Non-Ideal Clamped			

## Data Availability

Data is contained within the article.

## References

[1] Kandlikar S.G., Garimella S., Li D., Colin S., King M.R. (2006). Heat Transfer and Fluid Flow in Minichannels and Microchannels.

[2] Park S.K., Gao X.-L. (2006). Bernoulli–Euler beam model based on a modified couple stress theory. J. Micromech. Microeng..

[3] Ma H.M., Gao X.-L., Reddy J.N. (2008). A microstructure-dependent Timoshenko beam model based on a modified couple stress theory. J. Mech. Phys. Solids.

[4] Yang F., Chong A.C.M., Lam D.C.C., Tong P. (2002). Couple stress based strain gradient theory for elasticity. Int. J. Solids Struct..

[5] Atcı D., Bağdatlı S.M. (2017). Vibrations of fluid conveying microbeams under non-ideal boundary conditions. Microsyst. Technol..

[6] Younis M.I., Nayfeh A.H. (2003). A study of the nonlinear response of a resonant microbeam to an electric actuation. Nonlinear Dyn..

[7] Nayfeh A.H., Ouakad H.M., Najar F., Choura S., Abdel-Rahman E.M. (2010). Nonlinear dynamics of a resonant gas sensor. Nonlinear Dyn..

[8] Akkoca Ş., Bağdatlı S.M., Toğun N.K. (2021). Linear vibration motions of a microbeam supported from the middle. J. Fac. Eng. Archit. Gazi Univ..

[9] Akbaş Ş.D. (2012). Investigation of Post-Buckling Behavior of Functionally Graded Beams under Temperature Effect. Ph.D. Dissertation.

[10] Yildirim K. (2021). Vibration suppression of a micro beam subjected to magneto-electric load. Sigma J. Eng. Nat. Sci..

[11] Li S.R., Su H.D., Cheng C.J. (2009). Free vibration of functionally graded material beams with surface-bonded piezoelectric layers in thermal environment. Appl. Math. Mech..

[12] Warminska A., Manoach E., Warminski J. (2016). Vibrations of a composite beam under thermal and mechanical loadings. Procedia Eng..

[13] Hamza-Cherif R., Meradjah M., Zidour M., Tounsi A., Belmahi S., Bensattalah T. (2018). Vibration analysis of nano beam using differential transform method including thermal effect. J. Nano Res..

[14] Zhang P., Schiavone P., Qing H. (2022). Local/nonlocal mixture integral models with bi-Helmholtz kernel for free vibration of Euler-Bernoulli beams under thermal effect. J. Sound. Vib..

[15] Abouelregal A.E., Marin M. (2020). The response of nanobeams with temperature-dependent properties using state-space method via modified couple stress theory. Symmetry.

[16] Ghadiri M., Safarpoor N. (2017). Vibration of rotating functionally graded (FG) Timoshenko nanobeam with nonlinear thermal distribution. Mech. Adv. Mater. Struct..

[17] Mamu R.M., Togun N. (2024). Thermal effects on nonlinear vibration of nonlocal nanobeam embedded in nonlinear elastic medium. Acta Mech..

[18] Andrianov I.V., Awrejcewicz J., Koblik S.G., Starushenko G.A. (2024). Nonlinear oscillation of a microbeam due to an electric actuation—Comparison of approximate models. ZAMM J. Appl. Math. Mech..

[19] Eiadtrong S., Wattanasakulpong N., Vo T.P. (2023). Thermal vibration of functionally graded porous beams with classical and non-classical boundary conditions using a modified Fourier method. Acta Mech..

[20] Abouelregal A.E., Megahid S.F., Atta D., Al-Azmi A.M.K. (2024). Thermoelectric interactions in Euler-Bernoulli microbeams under the influence of a thermal pulse via the size-dependent couple stress model. Mech. Time-Depend. Mater..

[21] Chang W., Zhang P. (2025). Modeling of transverse vibration of non-uniform beams with elastic boundary conditions and thermal effect using two-phase local/nonlocal integral model. ZAMM–J. Appl. Math. Mech..

[22] Wu J.S., Chang B.H. (2013). Free vibration of axial-loaded multi-step Timoshenko beam carrying arbitrary concentrated elements using continuous-mass transfer matrix method. Eur. J. Mech..

[23] Lee J. (2013). Free vibration analysis of beams with non-ideal clamped boundary conditions. J. Mech. Sci. Technol..

[24] Kennedy M.C., O’Hagan A. (2001). Bayesian calibration of computer models. J. R. Stat. Soc. Ser. B (Stat. Methodol.).

